# Supplementary material for: Roving methyltransferases generate a mosaic epigenetic landscape and influence evolution in Bacteroides fragilis group
Source: Nat Commun. 2023 Jul 10;14:4082. doi: 10.1038/s41467-023-39892-6 (PMC10333322; doi:10.1038/s41467-023-39892-6)
Supplement: Supplementary file 2 — Supplementary Information [file 41467_2023_39892_MOESM2_ESM.pdf]

SUPPLEMENTARY INFORMATION

Roving methyltransferases generate a mosaic epigenetic landscape and influence evolution in *Bacteroides fragilis* group

Michael J. Tisza[1,a,*], Derek D. N. Smith[1,b,*], Andrew E. Clark[2,c], Jung-Ho Youn[2], NISC Comparative Sequencing Program[3], Pavel P. Khil[1,2], John P. Dekker[1,#]

[1]Bacterial Pathogenesis and Antimicrobial Resistance Unit, LCIM, NIAID, NIH, Bethesda, MD

[2]National Institutes of Health Clinical Center, National Institutes of Health, Bethesda, MD

[3]National Human Genome Research Institute, NIH, Bethesda, MD.

*Equal contribution

#Address correspondence to John Dekker: john.dekker@nih.gov

[a]Present address: Baylor College of Medicine, The Alkek Center for Metagenomics and Microbiome Research, Department of Molecular Virology and Microbiology, Houston, TX, USA

[b]Present address:Environment and Climate Change Canada, Ecotoxicology and Wildlife Health Division, Wildlife Toxicology Research Section, Ottawa, ON, CAN

[c]Present address: Department of Pathology, University of Texas Southwestern Medical Center, Dallas, TX, USA

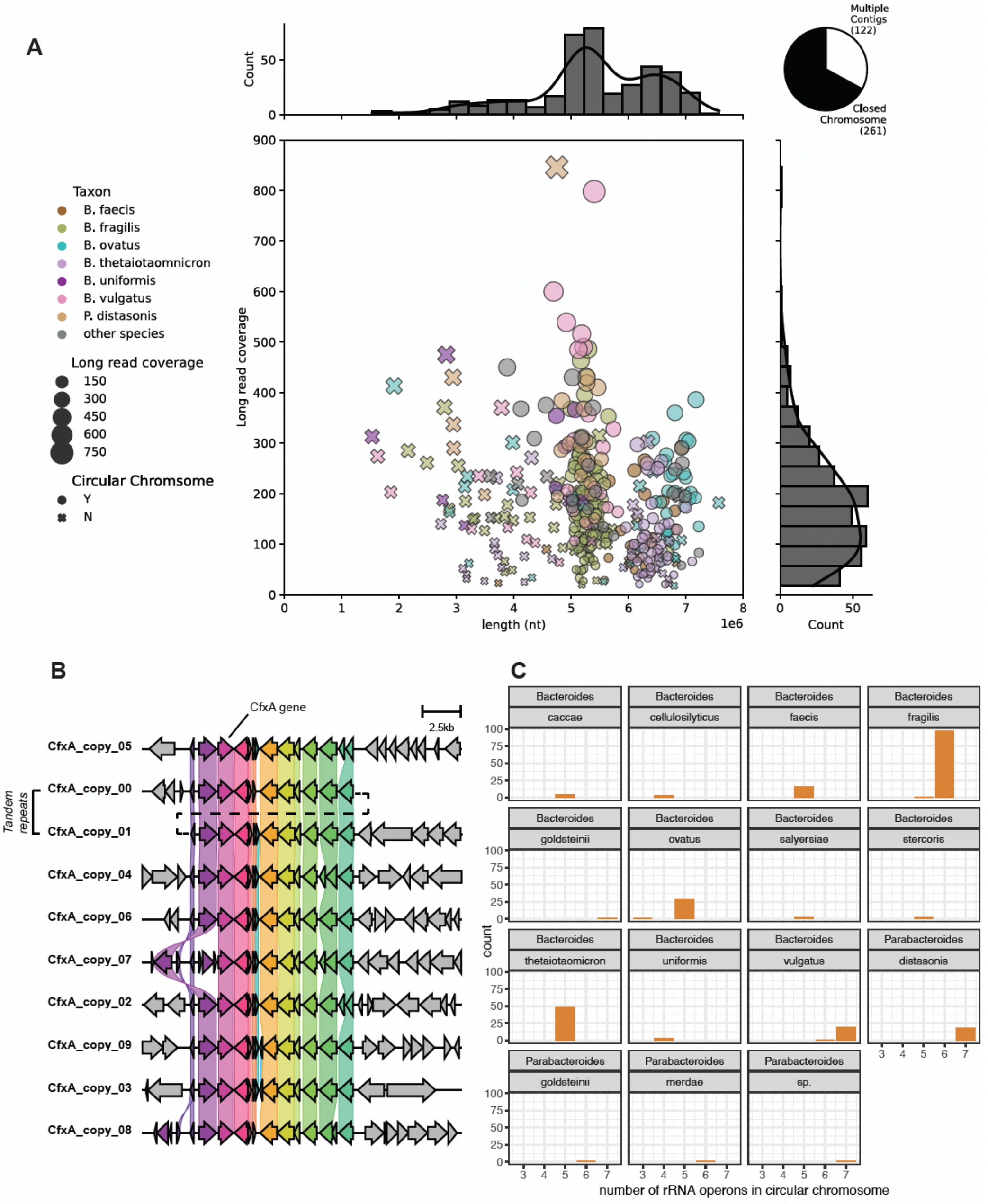

Supplementary Figure 1. Genome Assembly Summary. (A) Scatter plot demonstrating contiguity of 383 isolate genome assemblies. Each symbol represents a genome where the horizontal axis values indicate the length of the longest contig of the assembly and the vertical axis values indicate the coverage of this contig. Filled circles represent chromosomes that assembled as closed contigs with Flye, and crosses represent linear chromosome contigs or fragments. (B) An example of assembly through long, multicopy repeats (Tn4555 carrying cfxA beta-lactamase) from genome of BFG-488 (*B. fragilis*). (C) Number of distinct rRNA operons identified in all genomes for which the chromosome was assembled as a complete circle.

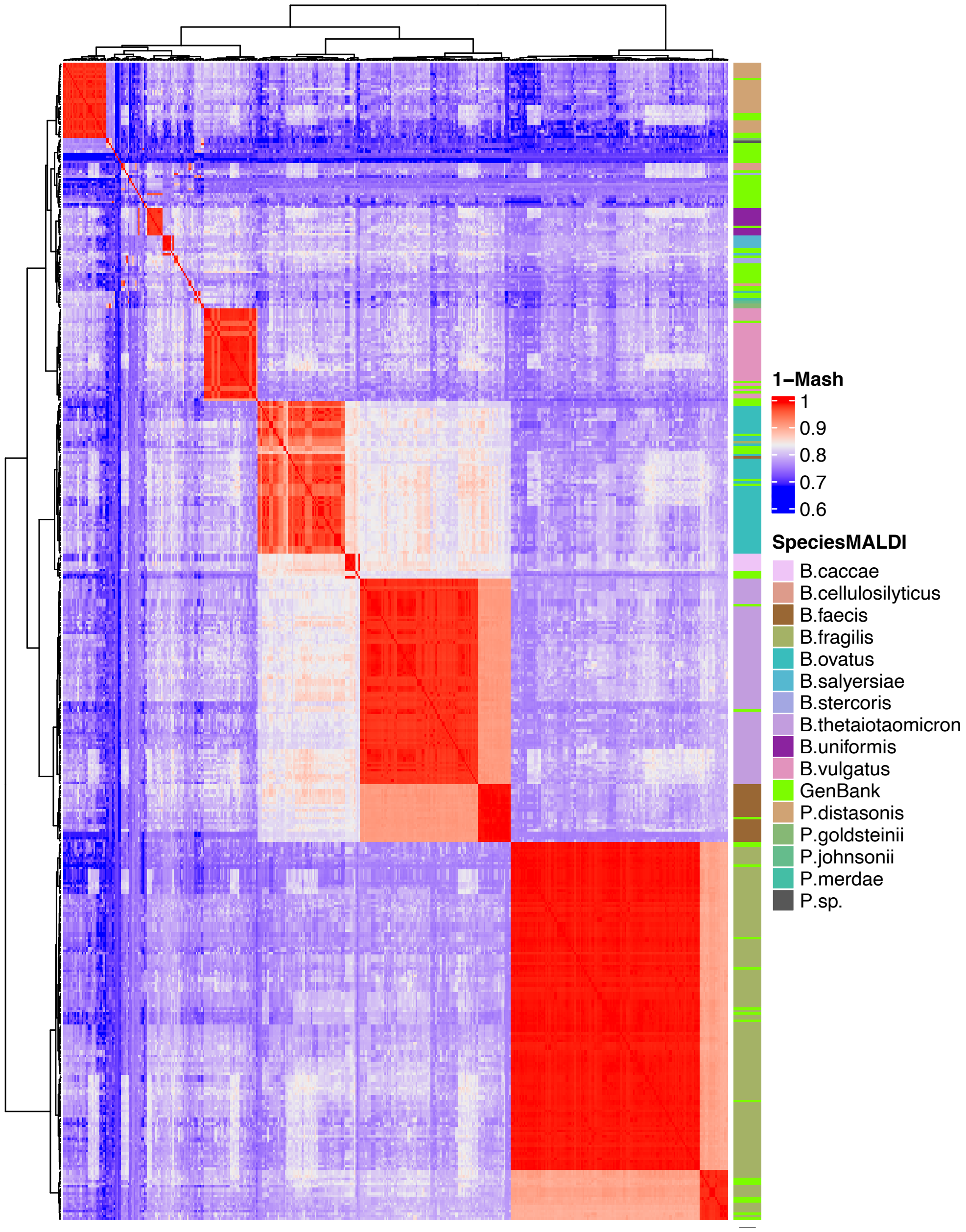

Supplementary Figure 2. Comparison of Genome Assemblies and GenBank Isolates. Comparison of genomes of 383 isolates sequenced in this study and 79 *Bacteroides fragilis* group representatives from GenBank presented as a hierarchically-clustered matrix of all-versus-all 1-Mash scores. The color bar at the right represents MALDI or GenBank species designations for each organism.

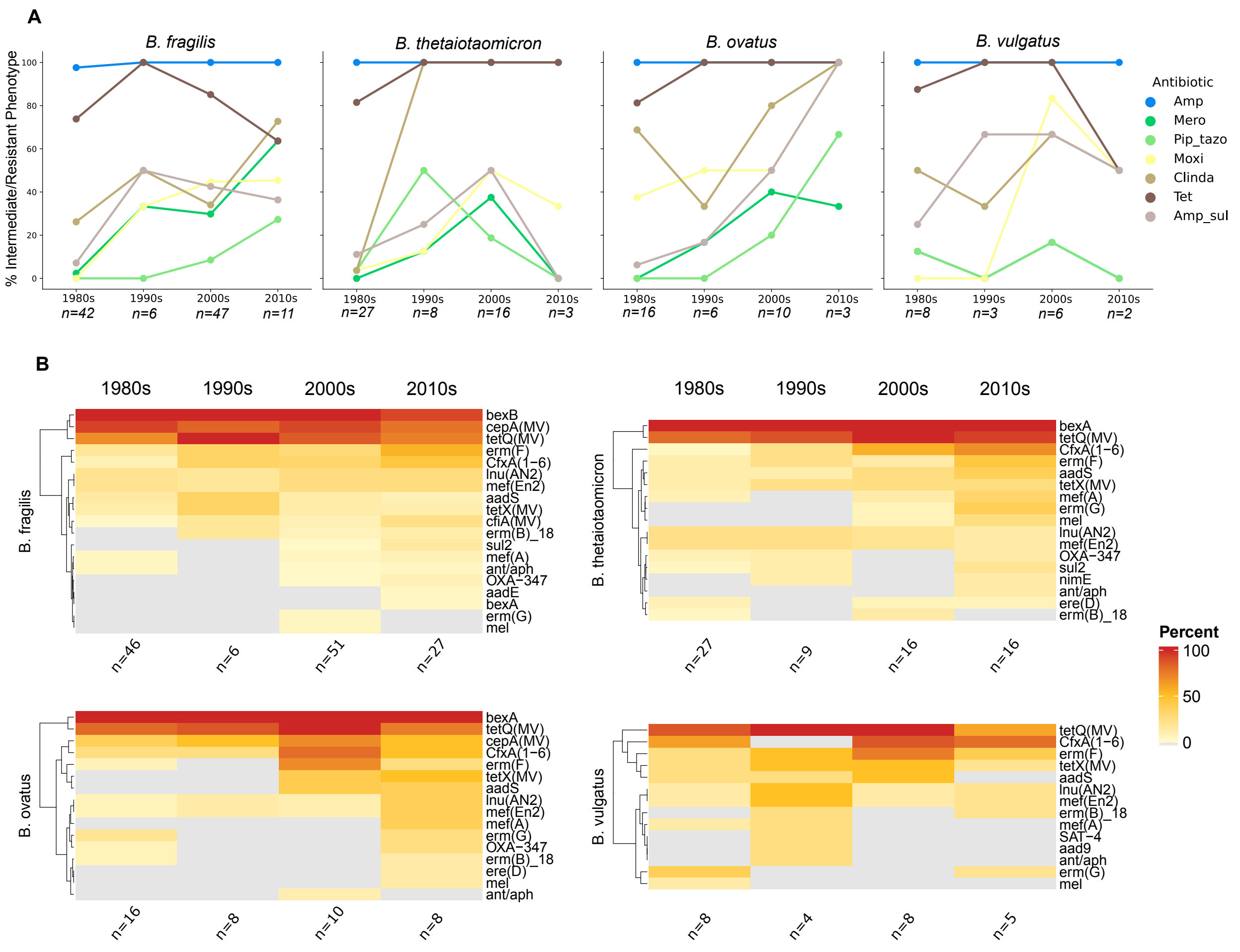

Supplementary Figure 3. Antimicrobial Resistance Phenotype and Genotype of clinical isolates across decades. (A) Change in aggregate percentages of isolates testing intermediate or resistant to antibiotics indicated at the right. Data are reported for four tested species. Number of included isolates in each decade is given under the horizontal axis label. (B) A heatmap of percentages of isolates within each species at each time point containing at least one representative of the indicated AMR gene. AMR genes were detected using Abricate. The denominator is total number of isolates from the given species in each decade. Annotation and allelic variants of tetQ/tetX (tetracycline resistance), ant/aph (aminoglycoside resistance), and cfxA 1-6 (beta lactam resistance) were individually clustered and counted as a group in the proportion calculations.

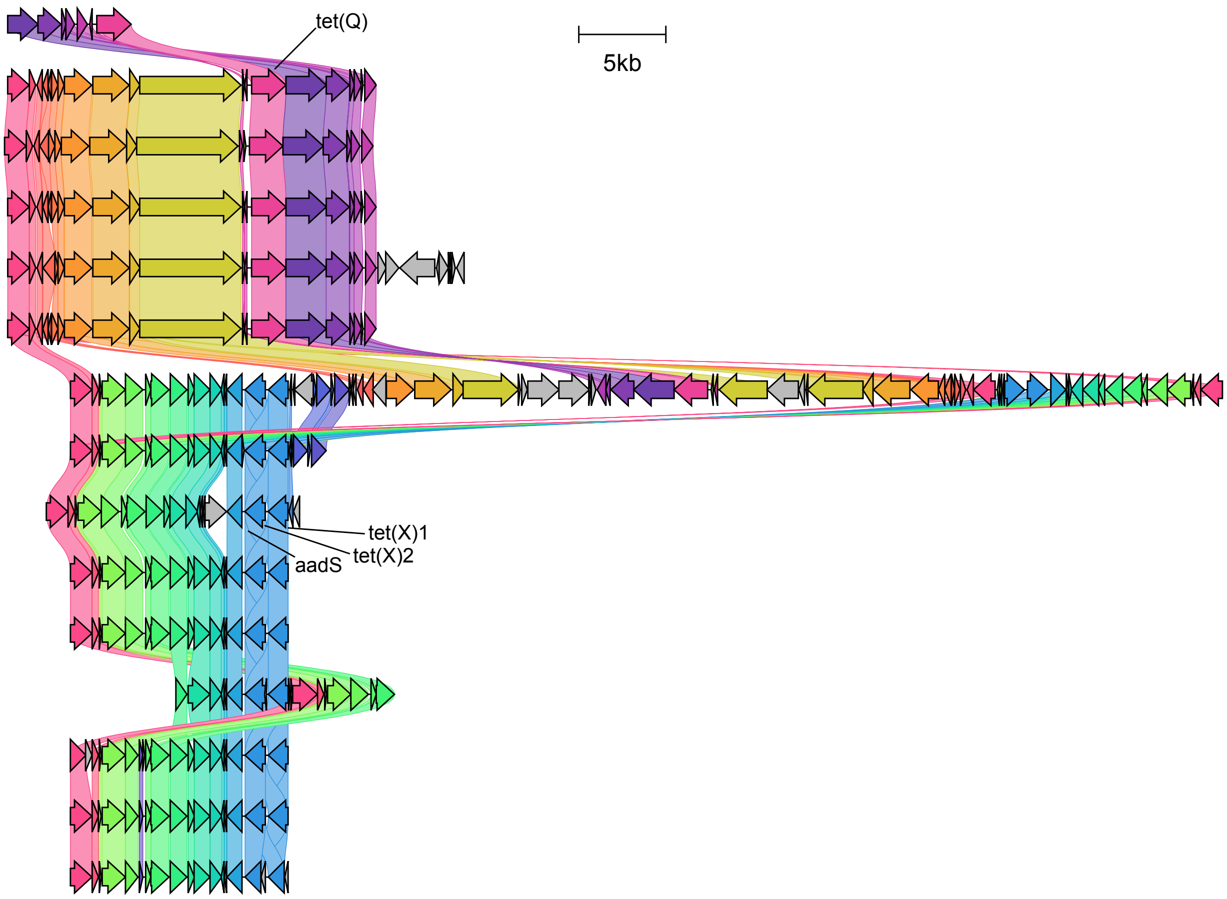

Supplementary Figure 4. Accessory regions with alignments to 12 BFG species. Clinker illustration of prokka annotation of accessory regions with alignments in 12 BFG species. Alignments were made only between accessory regions of genomes. Antibiotic resistance genes are labeled.

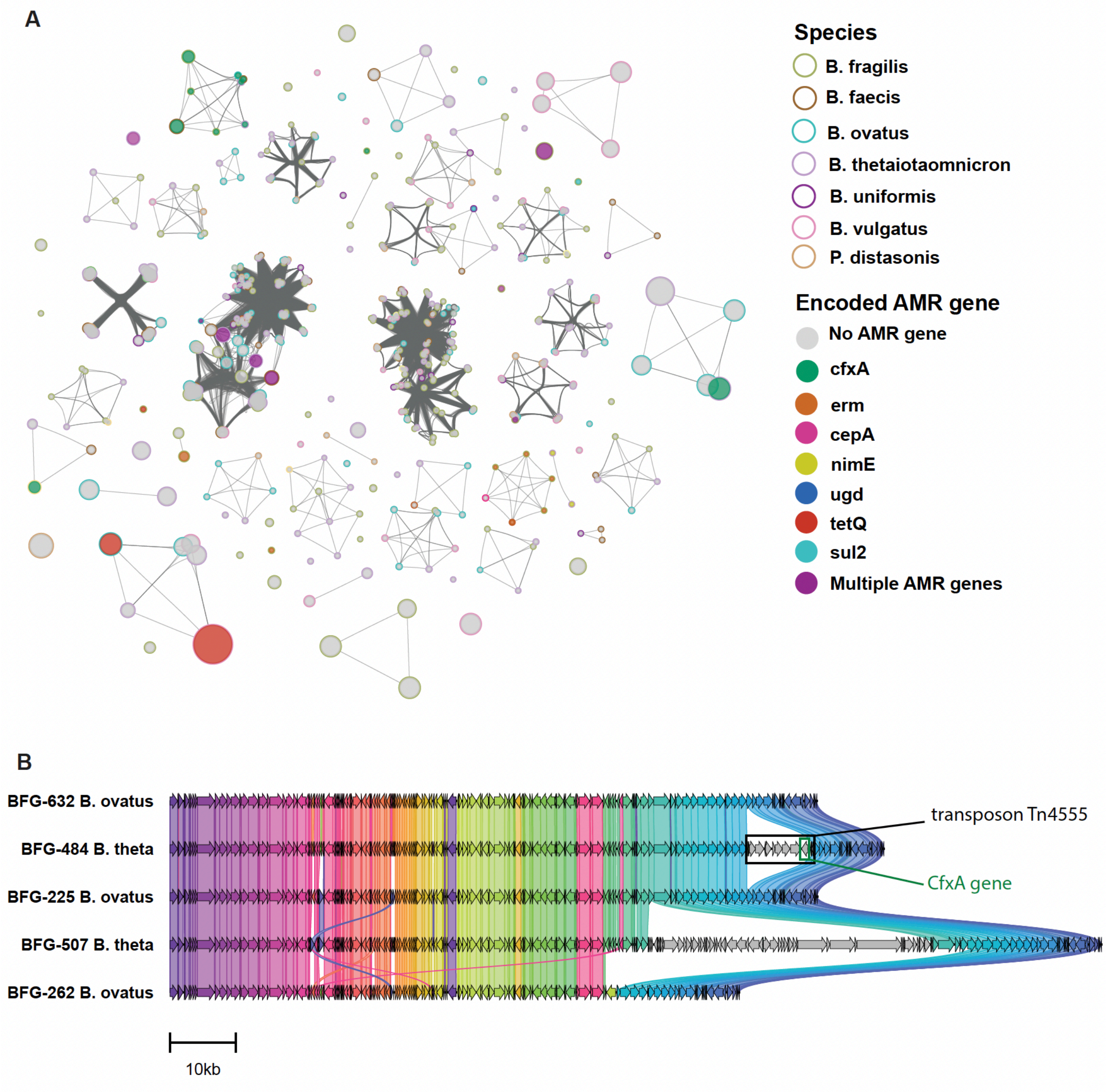

Supplementary Figure 5. Episome/Plasmid analysis of BFG isolate genomes. (A) Network plot in which each episome/plasmid is represented as a node indicated by a filled circle. The area of the circle corresponds to the length of the sequence. Edges connect nodes within the same cluster (95% ANI, 85% AF). (B) Clinker diagrams of plasmids within a cluster. Insertion of transposon 4555 is labeled.

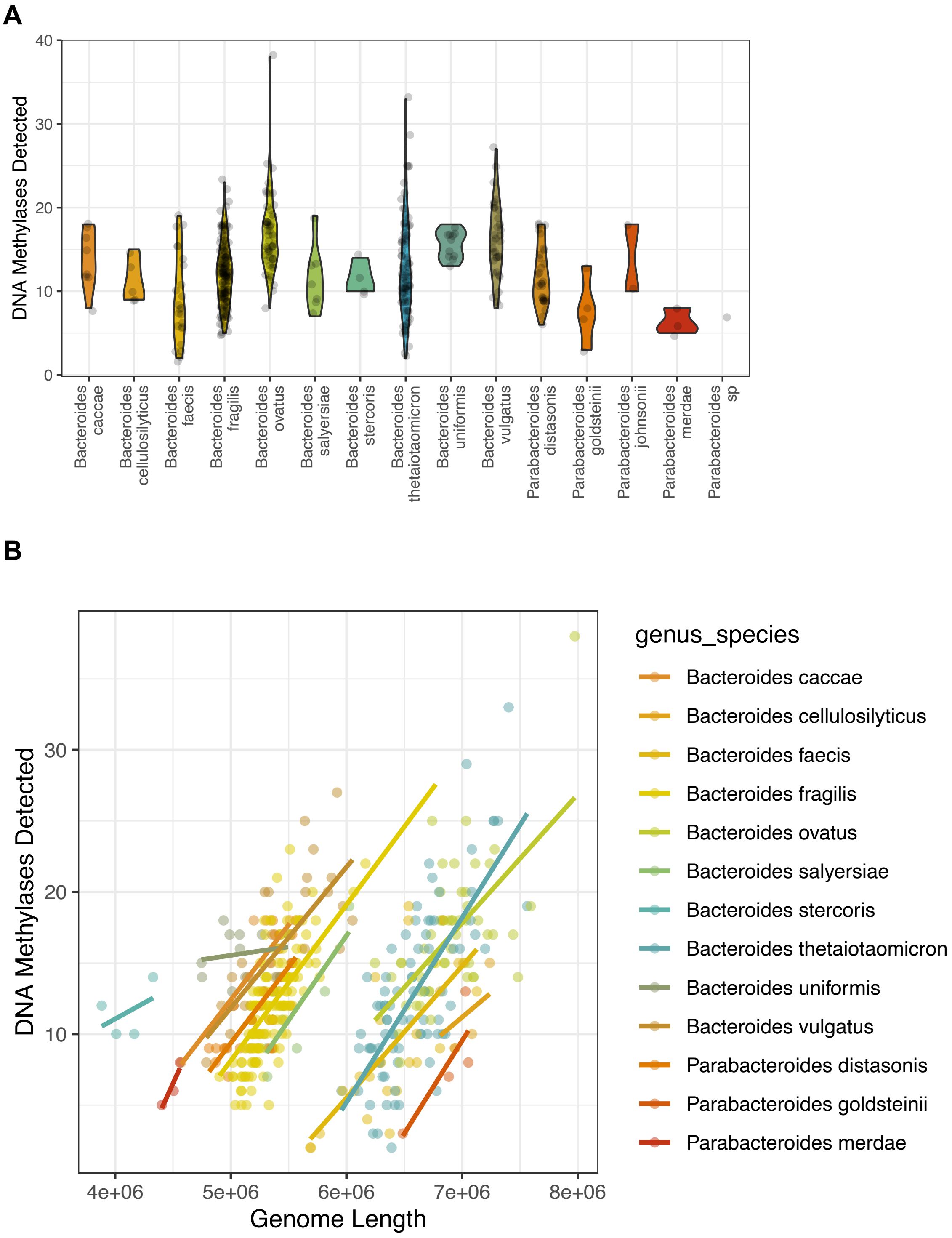

Supplementary Figure 6. DNA methyltransferase gene number generally correlates with genome length within each species. (A) Distribution of putative methyltransferase genes identified by DNA Methylase Finder for each species. (B) Correlations of detected DNA methyltransferases and species genome length.

A

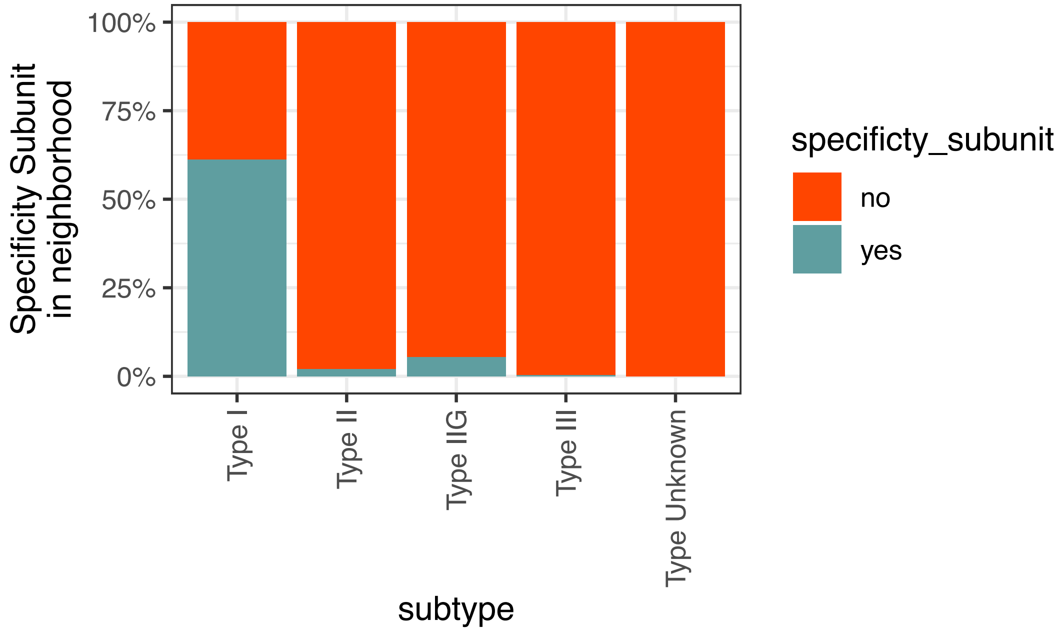

B

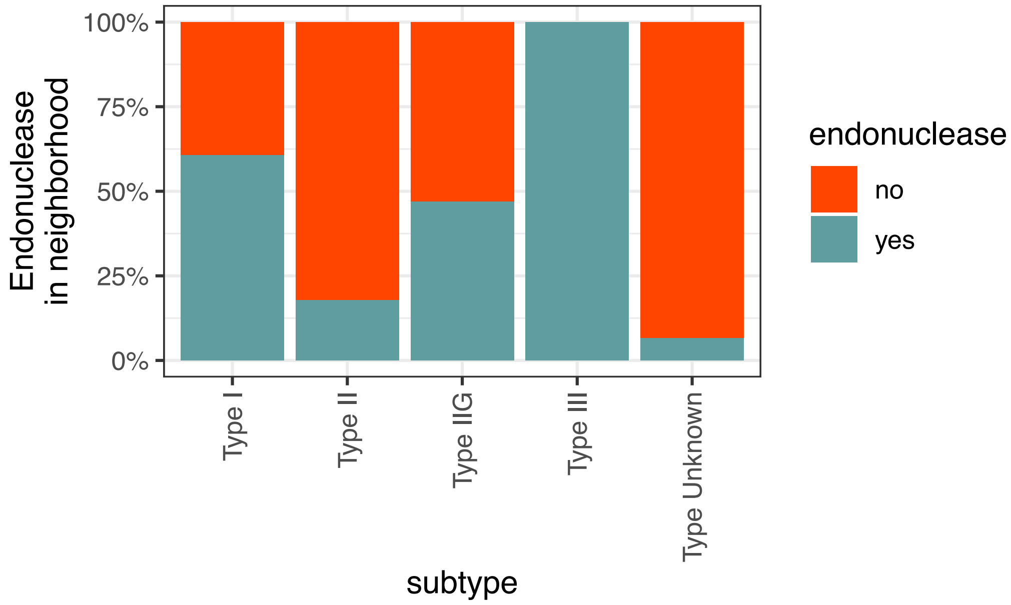

Supplementary Figure 7. DNA methyltransferase neighborhood analysis. (A) Summary of specificity subunits in the neighborhood of different putative subtypes of DNA methyltransferases identified in the BFG set. (B) Summary of restriction endonucleases in the neighborhood of different putative subtypes of DNA methyltransferases identified in the BFG set.

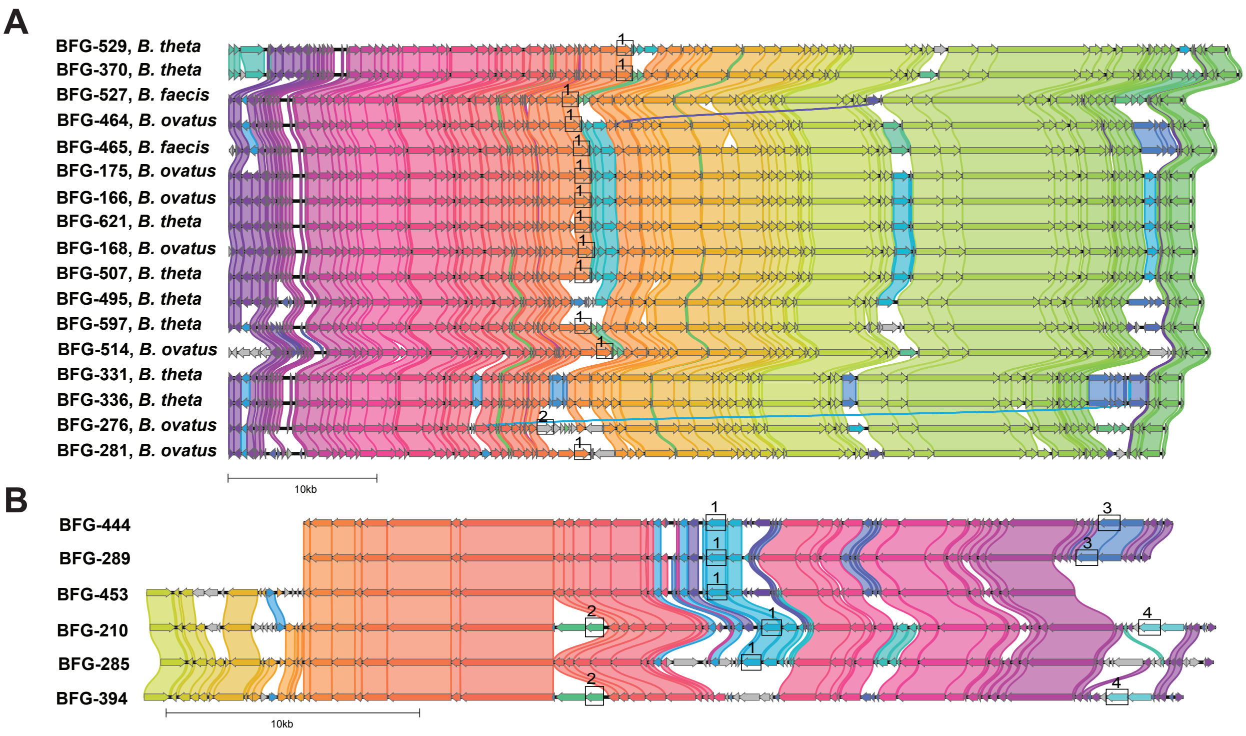

Supplementary Figure 8. DNA methyltransferase gene diversity in two BFG phage genome clusters. (A and B) Genomes from the same vOTUs (95% ANI, 85% AF) with DNA methyltransferase genes annotated with a black box and number corresponding to gene family (numbers are arbitrary).

Example 1: *B. fragilis* isolate BFG-420 in which **CTCAT** and **CCAGT** motifs are merged.

Step 1: Inspect motif chart from *Nanodisco motif* command, **CYSAK**.

- Note that there are many dots at the baseline in every position.
- This is not consistent with the expectation that the majority of motifs should contain the methylated base.
- A pattern with moderate peaks at '7' and '9' and higher peak at '8' becomes apparent for **CTCAT** motif.

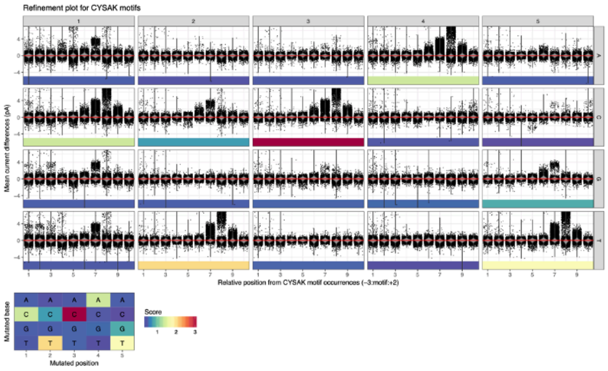

Step 2: Now manually try this motif identified by manual inspection. In this case, **CTCAT.**

- This motif has substantial signal difference at the majority of motif loci. Thus, it is a high-confidence motif.
- But what about that other signal pattern buried within **CYSAK**?

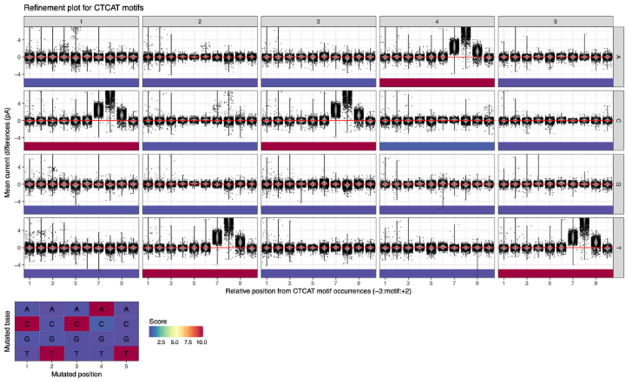

Step 3: Run the *Nanodisco motif* command again to iterate over the data while ignoring **CTCAT**.

- The motif **CCAGT** has been returned, which has the second peak pattern that was visible in the original **CYSAK**.
- We can conclude that *Nanodisco motif* incorrectly merged these motifs based on the "**CA**" in the middle of each motif.
- The two higher confidence motifs are **CCAGT** and **CTCAT**

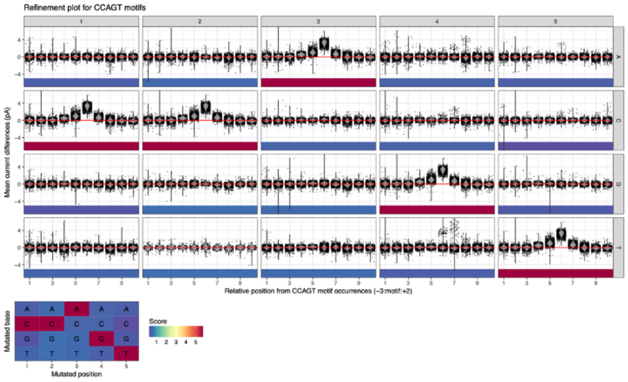

Supplementary Figure 9. Procedure for manual identification of merged methylation motifs in Nanodisco output and correction.

<u>Example 2:</u> *B. thetaiotaomicron* BFG-484 motif missing initial discriminatory G nucleotide.

<u>Step 1</u>: Inspect motif chart from *Nanodisco motif* command, **GCANNNNNNNRTTT**.

- There are many dots that are at the baseline in every position.
- However, there are not 2 distinct signals that emerge.
- This is how a Nanodisco motif is returned that is "too short" and missing a discriminatory end nucleotide.

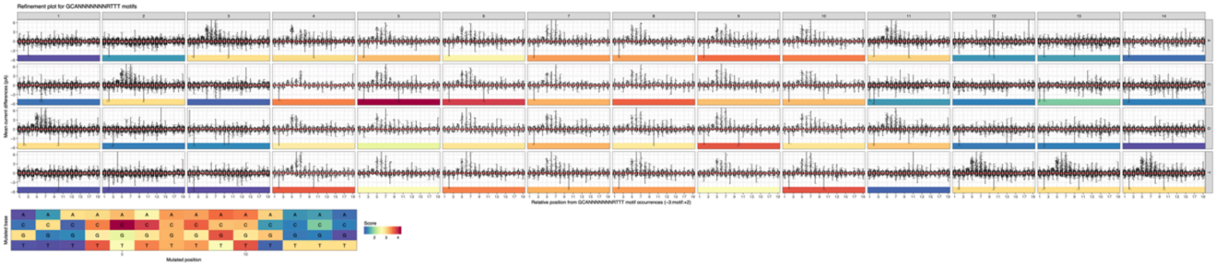

<u>Step 2:</u> Extend the motif with **<u>N</u>**<u>'s</u> on either end, i.e. **NGCANNNNNNNRTTTN**

- It now becomes obvious that the "**G**" added to the beginning of the motif results in high quality identification at this position. Note that the nucleotides at the end of the motif are all equal.

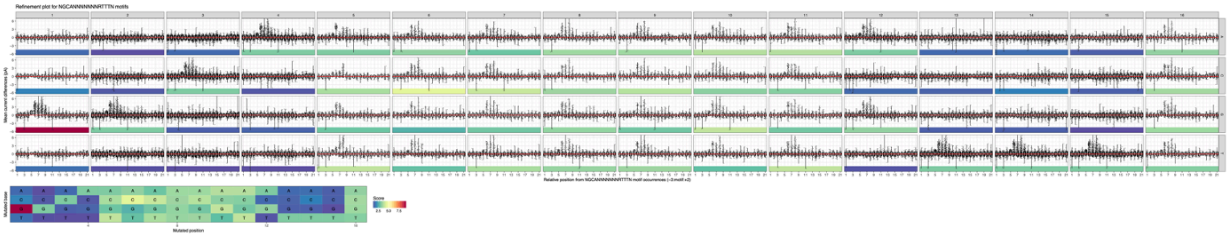

<u>Step 3:</u> Try the original motif plus **G** at the beginning, <u>i.e.</u> **GGCANNNNNNNRTTT**

- The many dots at baseline seen in the original output at the top have now disappeared, and we have identified a motif with much higher confidence.
- Note that it may still seem a little noisy, but if you look at the 5th position, a few distinct, but fully penetrant patterns are clear.

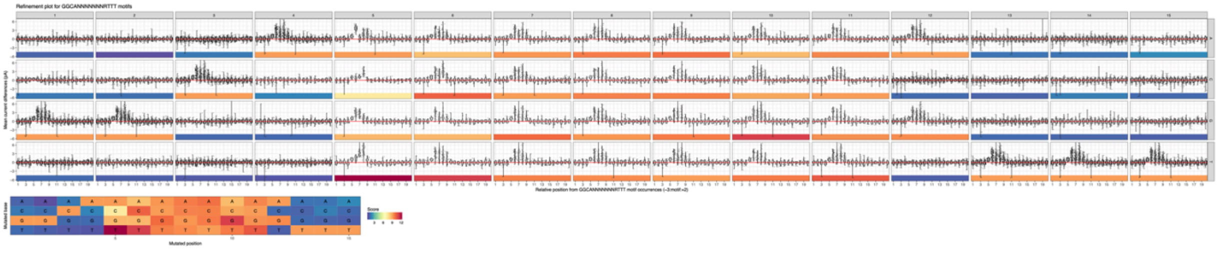

Supplementary Figure 10. Procedure for manual identification of truncated methylation motifs in Nanodisco output and correction.

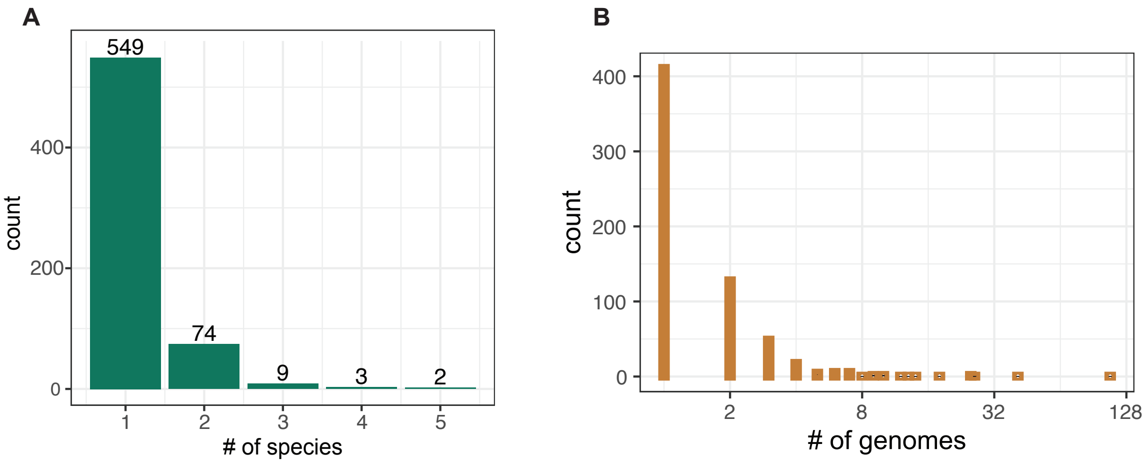

Supplementary Figure 11. DNA methylation motifs distribution across genomes and species. (A) Number of species each motif was detected in. Most motifs were detected in a single species. (B) Number of genomes each motif was detected in. Most motifs were detected only in a single genome.

Supplementary Figure 12A-D (below). Sequence analysis demonstrating distribution of DNA methylation motifs across AMR genes and promoters in *B. fragilis*. (5A-D) Dereplicated AMR genes and promoters were extracted from each indicated species, and species-specific DNA methylation motifs that were detected in this study were mapped to these sequences. The horizontal grey dotted line marks the start codon of the genes, black diamonds mark the stop codons, blue rectangles mark the conserved *Bacteroides* strong promoter motif "TAnnTTTG". All DNA methylation motif matches are binned along the length of the analyzed genomic segment, and vertical grey bars represent the number of motifs in a given bin. Continuous envelopes are fit to tops of bars. Positive and negative strands are colored as indicated in the legend.

Supplementary Figure 12A. Sequence analysis demonstrating tiling of DNA methylation motifs across AMR genes and promoters in *B. fragilis sensu stricto*.

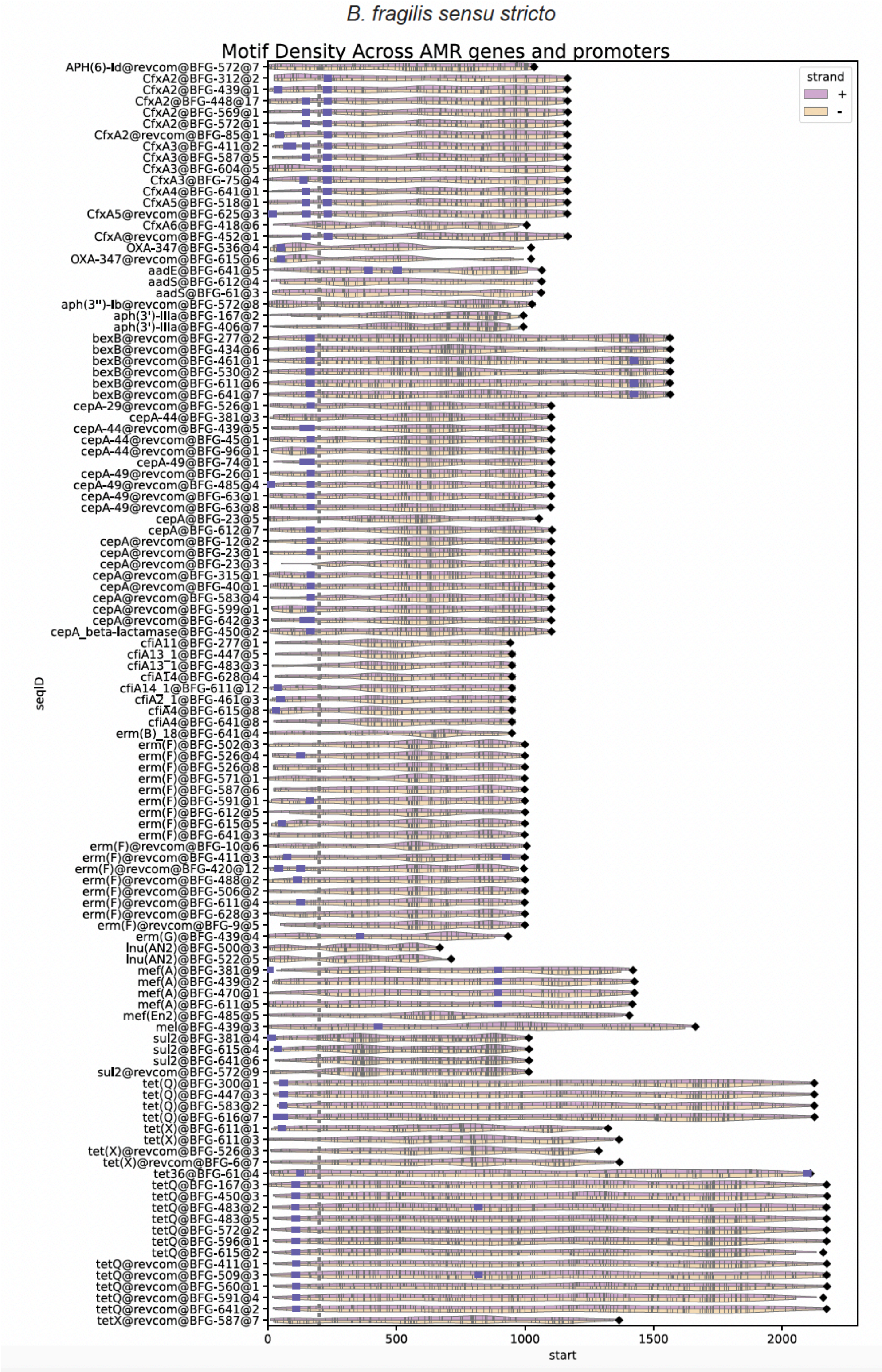

Supplementary Figure 12B. Sequence analysis demonstrating tiling of DNA methylation motifs across AMR genes and promoters in *B. ovatus*.

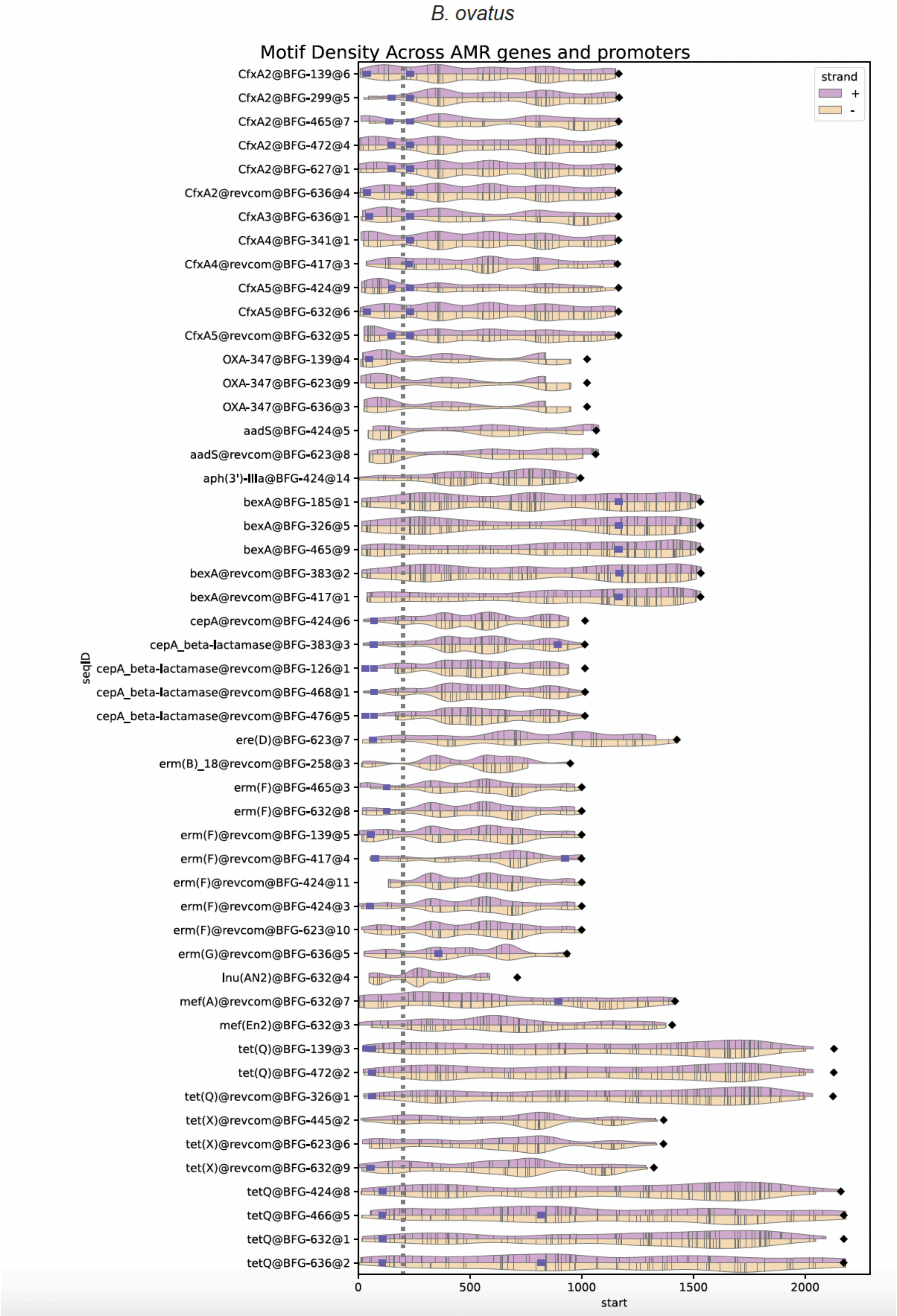

Supplementary Figure 12C. Sequence analysis demonstrating tiling of DNA methylation motifs across AMR genes and promoters in *B. thetaiotamicron*

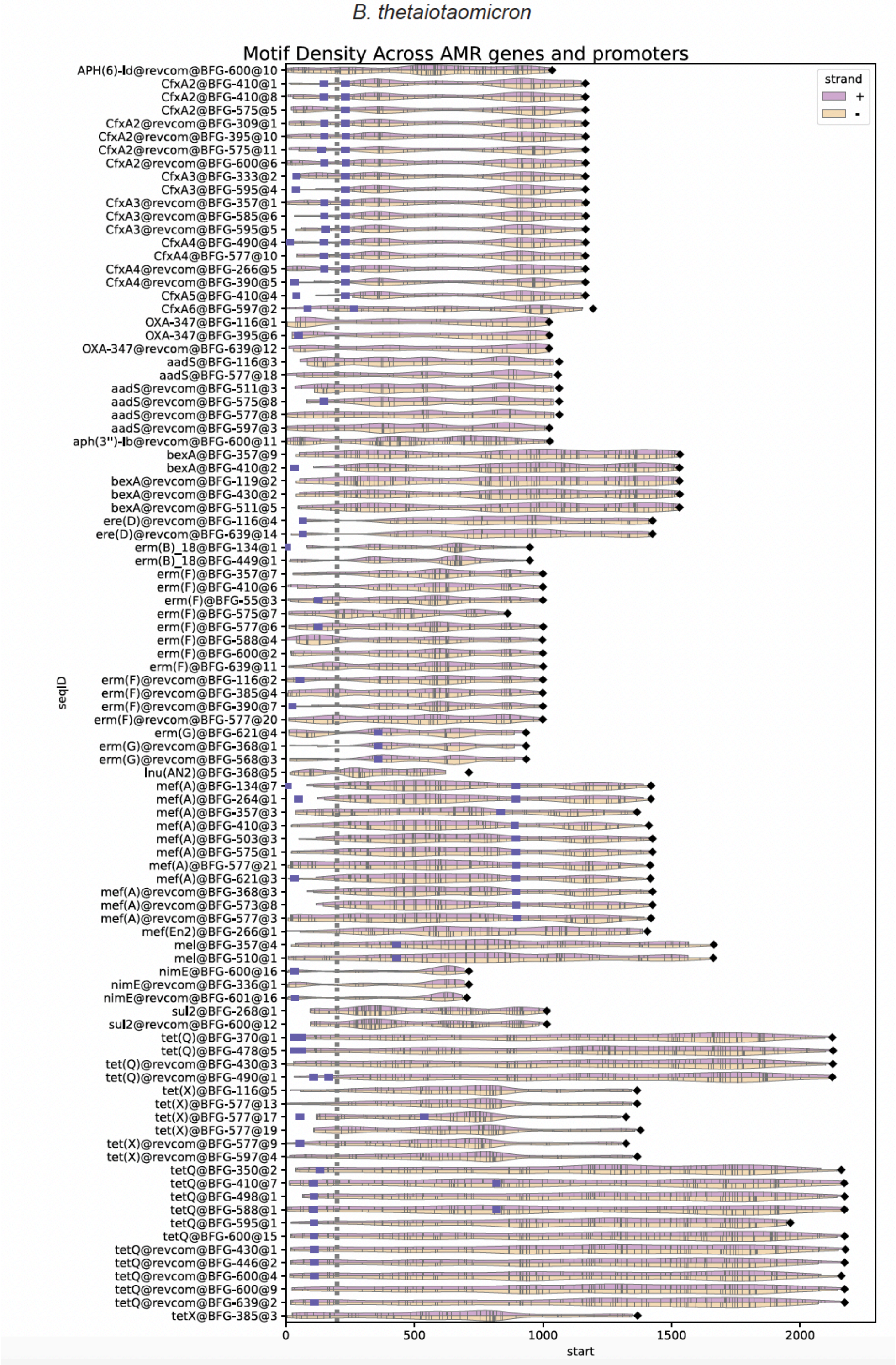

Supplementary Figure 12D. Sequence analysis demonstrating tiling of DNA methylation motifs across AMR genes and promoters in *P. distasonsis*

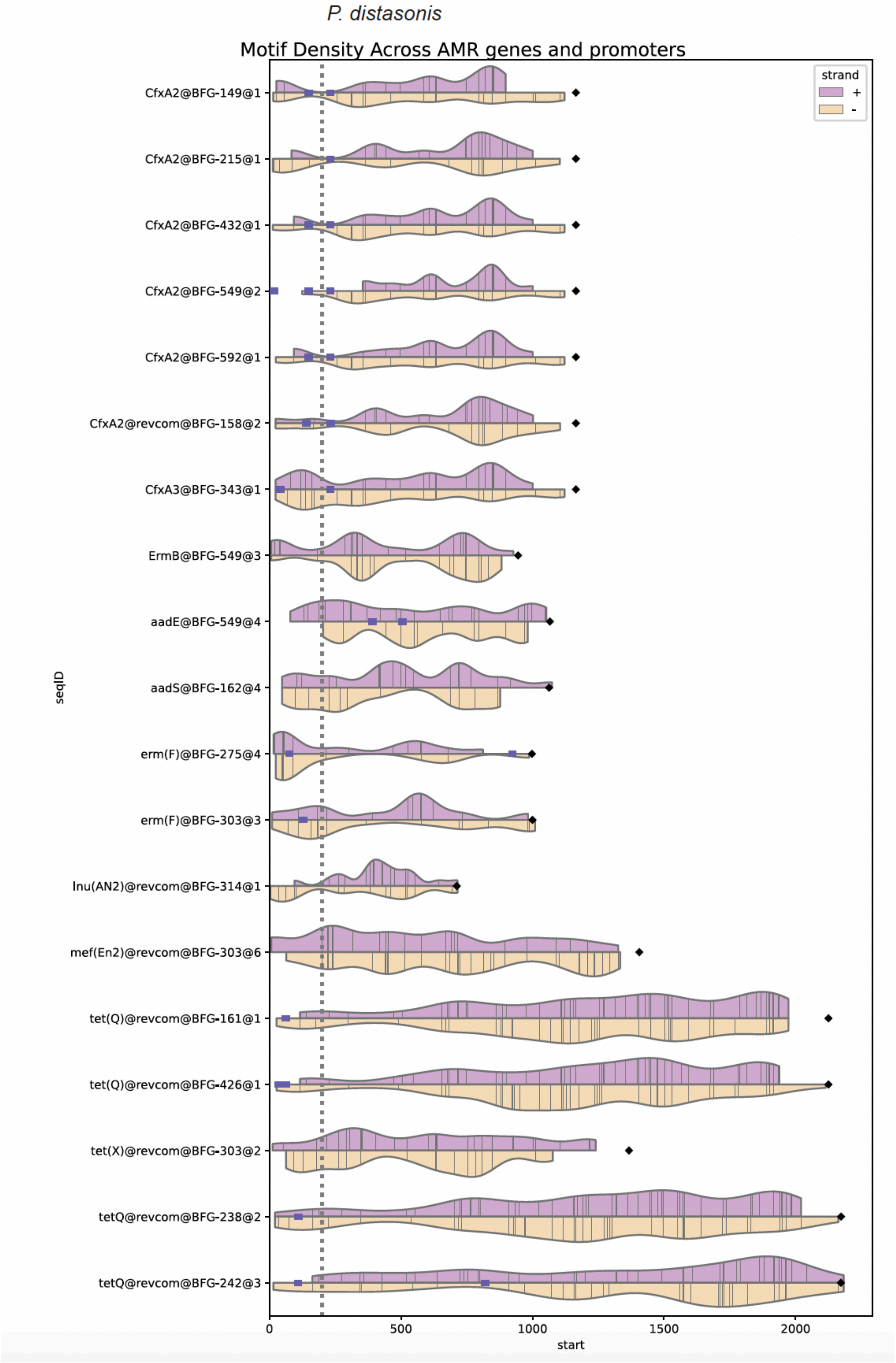

Supplementary Figure 12E. Sequence analysis demonstrating tiling of DNA methylation motifs across AMR genes and promoters in *B. vulgatus.*

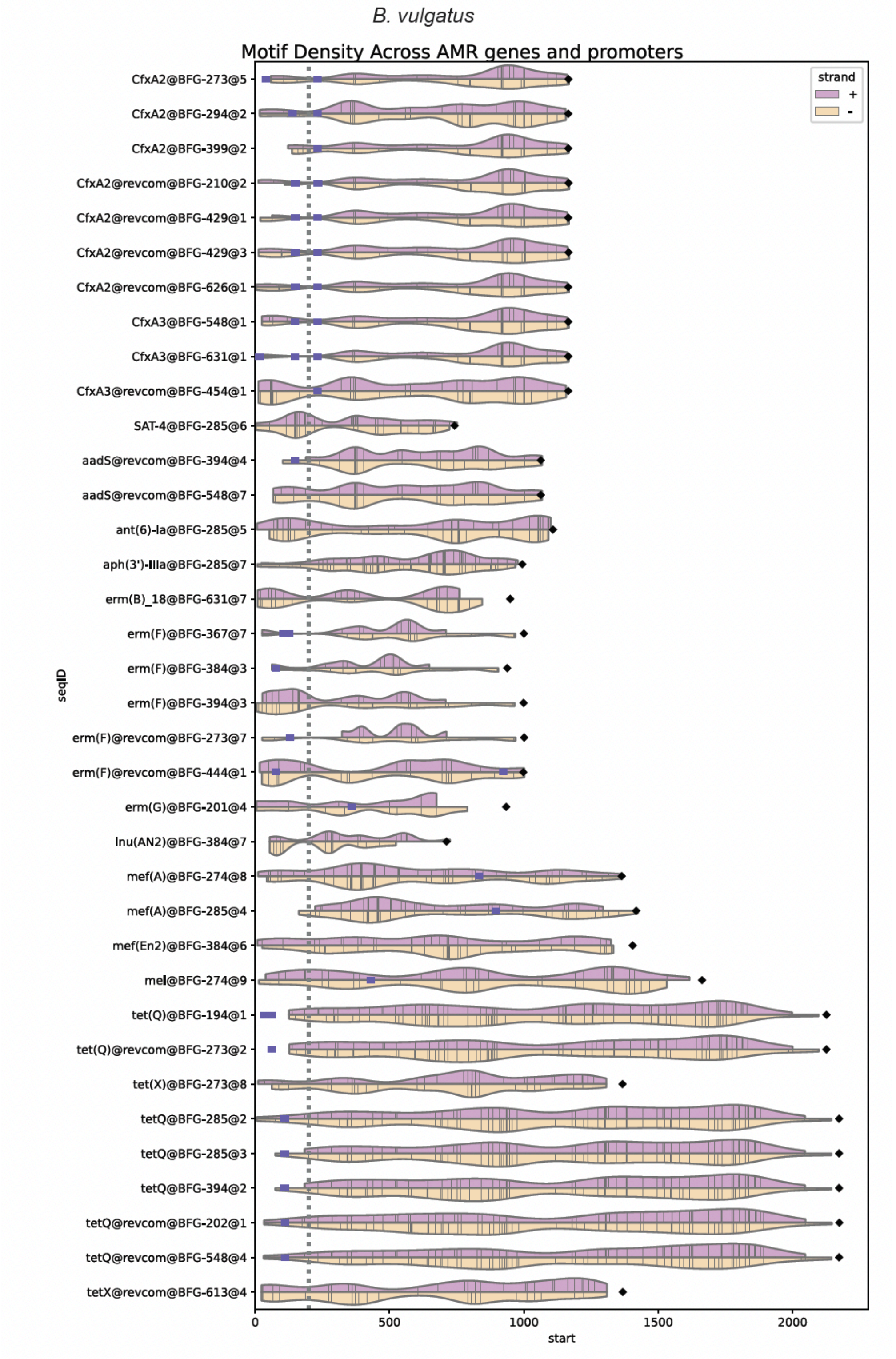

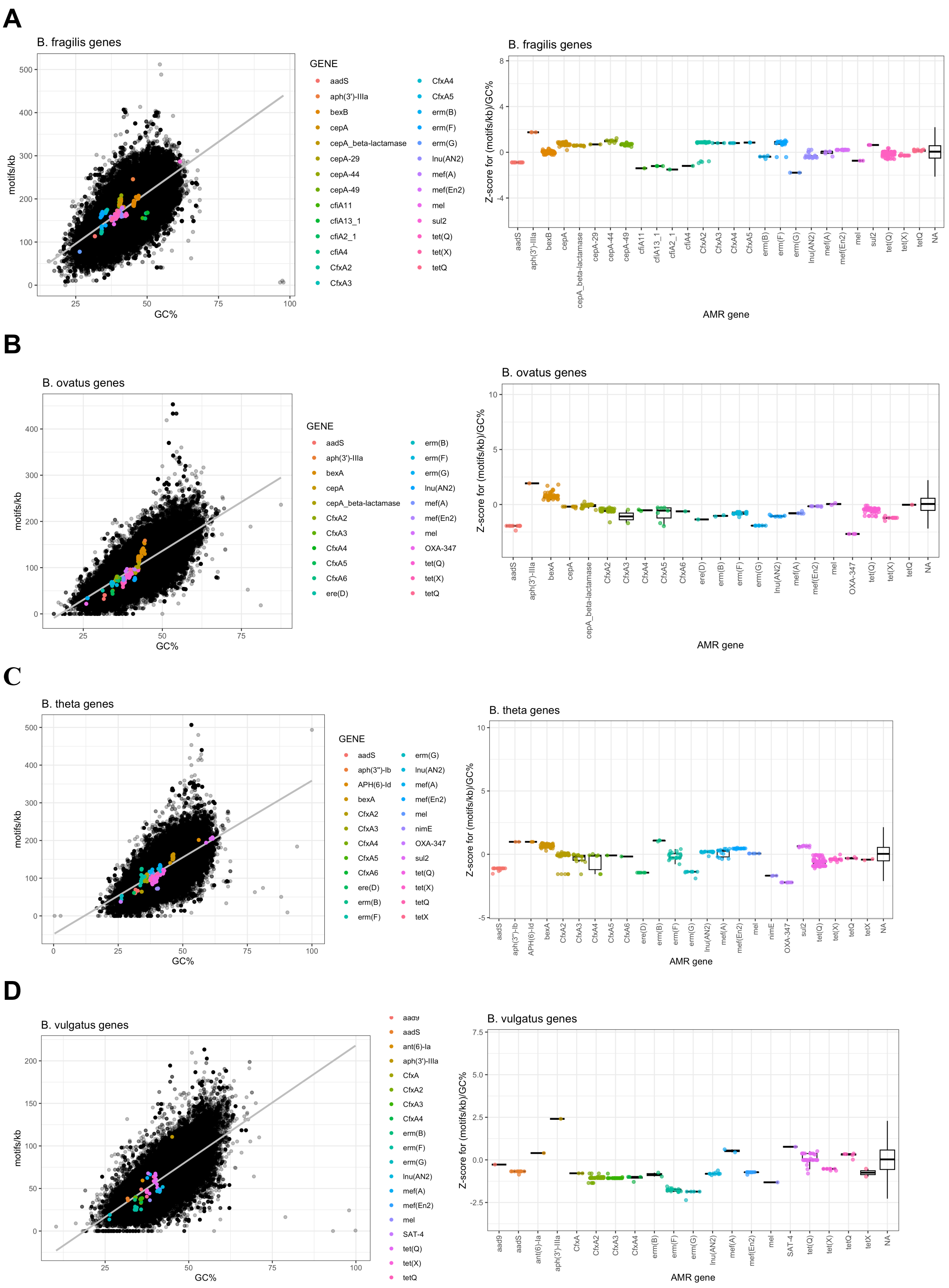

A
B. fragilis genes
motifs/kb
GC%
GENE
aadS
aph(3')-IIIa
bexB
cepA
cepA_beta-lactamase
cepA-29
cepA-44
cepA-49
cfiA11
cfiA13_1
cfiA2_1
cfiA4
CfxA2
CfxA3
CfxA4
CfxA5
erm(B)
erm(F)
erm(G)
lnu(AN2)
mef(A)
mef(En2)
mel
sul2
tet(Q)
tet(X)
tetQ
B. fragilis genes
Z-score for (motifs/kb)/GC%
AMR gene
NA
B
B. ovatus genes
motifs/kb
GC%
GENE
aadS
aph(3')-IIIa
bexA
cepA
cepA_beta-lactamase
CfxA2
CfxA3
CfxA4
CfxA5
CfxA6
ere(D)
erm(B)
erm(F)
erm(G)
lnu(AN2)
mef(A)
mef(En2)
mel
OXA-347
tet(Q)
tet(X)
tetQ
B. ovatus genes
Z-score for (motifs/kb)/GC%
AMR gene
NA
C
B. theta genes
motifs/kb
GC%
GENE
aadS
aph(3'')-Ib
APH(6)-Id
bexA
CfxA2
CfxA3
CfxA4
CfxA5
CfxA6
ere(D)
erm(B)
erm(F)
erm(G)
lnu(AN2)
mef(A)
mef(En2)
mel
nimE
OXA-347
sul2
tet(Q)
tet(X)
tetQ
tetX
B. theta genes
Z-score for (motifs/kb)/GC%
AMR gene
NA
D
B. vulgatus genes
motifs/kb
GC%
aad9
aadS
ant(6)-Ia
aph(3')-IIIa
CfxA
CfxA2
CfxA3
CfxA4
erm(B)
erm(F)
erm(G)
lnu(AN2)
mef(A)
mef(En2)
mel
SAT-4
tet(Q)
tet(X)
tetQ
tetX
B. vulgatus genes
Z-score for (motifs/kb)/GC%
AMR gene
NA

E

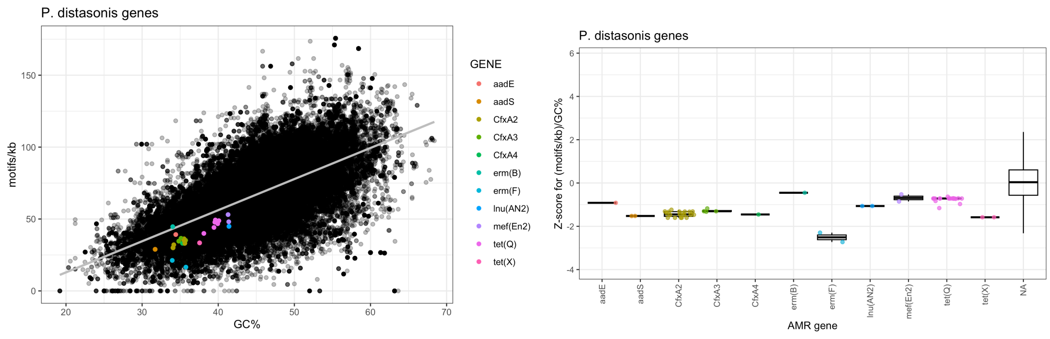

Supplementary Figure 13. Motif density in AMR genes compared to non-AMR genes. Genes from isolate genomes were extracted and methylation motifs found in one or more genomes of each species were mapped to corresponding genes. Antimicrobial resistance genes were called using abricate and the motif density of each gene was normalized by percentage GC content. Z-score was calculated and data were plotted by AMR gene category (aggregate non-AMR genes = “NA”). Lines represent iteratively reweighted least squares linear fits of all data in each plot. (A) *B. fragilis,* n = 108 genomes; (B) *B. ovatus*, n =44 genomes; (C) *B. thetaiotaomicron*, n = 54 genomes; (D) *B. vulgatus*, n = 28 genomes; (E) *P. distasonis*, n = 24 genomes. (All) Boxplot boxes show data quartiles and whiskers show 1.5X IQR.

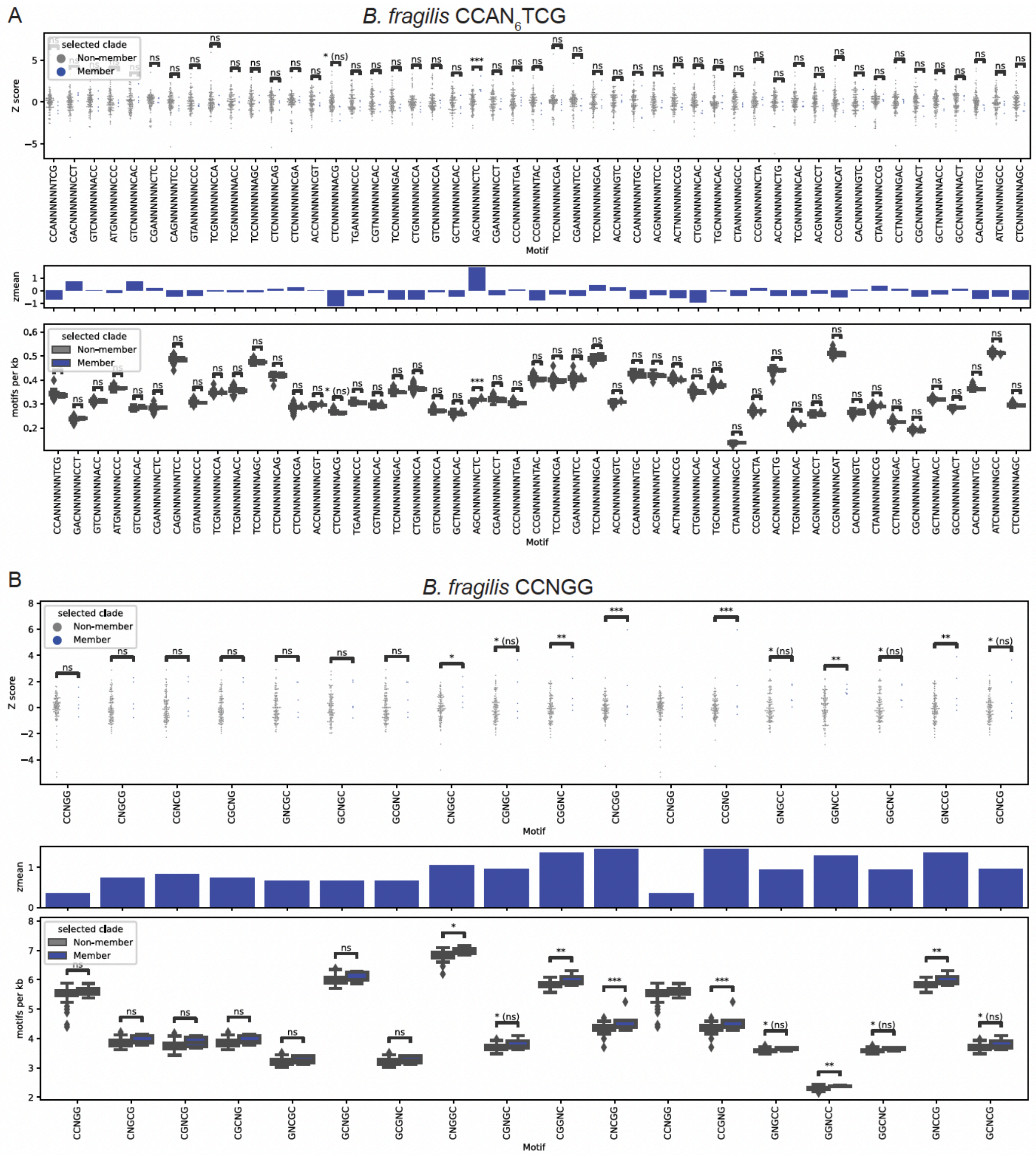

Supplementary Figure 14. Comparison of lineage-specific motif depletion to control motifs, *B. fragilis* CCANNNNNNTCG and *B. fragilis* CCNGG. (A) *B. fragilis* genomes (n = 108 genomes) were scanned for abundance of motif CCANNNNNNTCG and control motifs with the same base composition in non-member isolates (left) and member isolates (right) in each pair. Statistics on top and bottom panels show two-sided t-tests with Benjamini-Hochberg correction (1% FDR). Unadjusted p values are reported as follows: *: p <= 0.05, **: p <= 0.01, ***: p <= 1e-3, ****: p <= 1e-4; “ns” indicates that the reported p value was determined to be non-significant after testing with Benjamini-Hochberg at FDR = 1%. Middle panel is the mean Z score of the selected clade/lineage. (B) Similar to (A) but with *B. fragilis* and motif CCNGG. (All) Boxplot boxes show data quartiles and whiskers show 1.5X IQR with outliers drawn as diamonds.

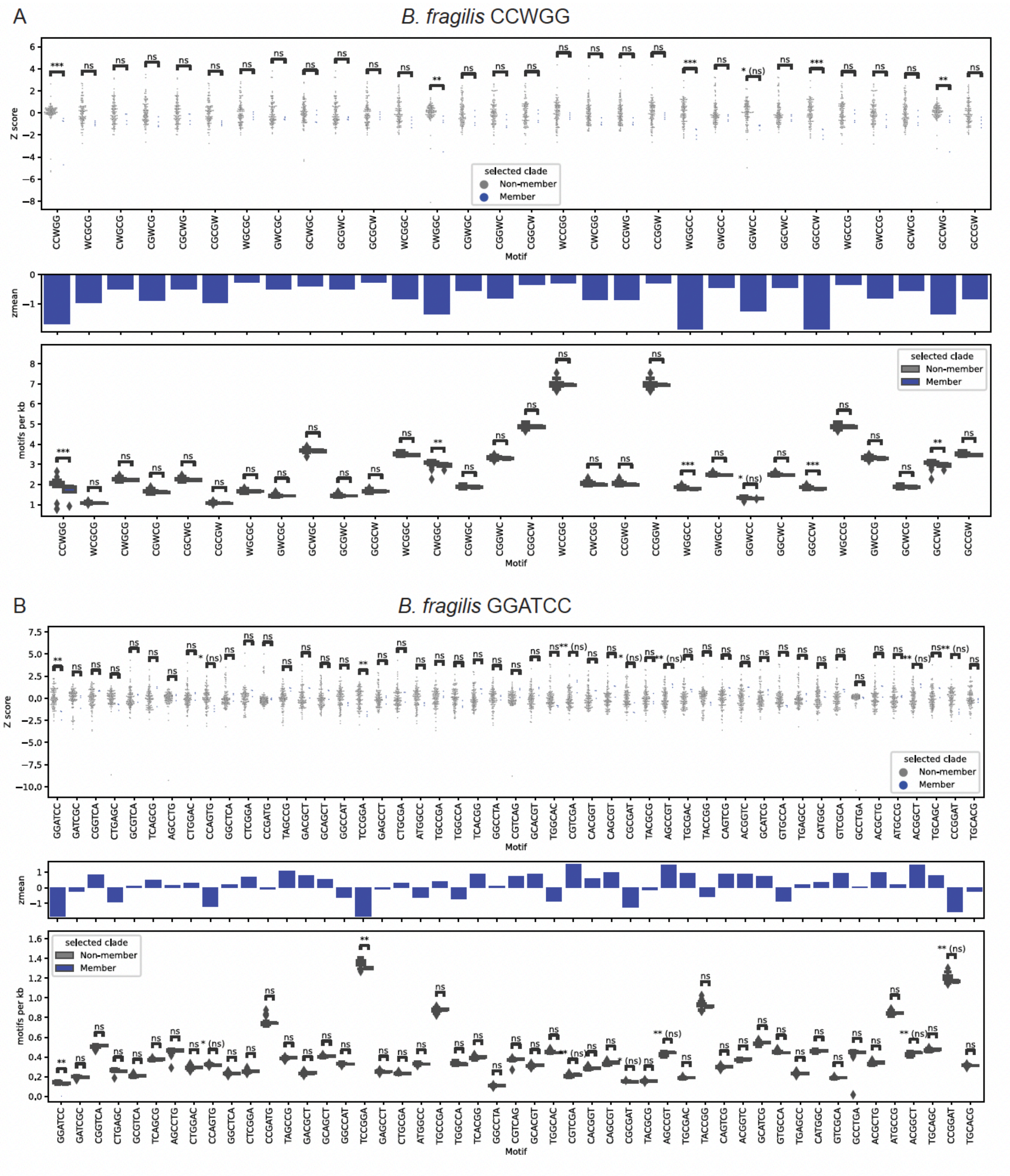

Supplementary Figure 15. Comparison of lineage-specific motif depletion to control motifs, *B. fragilis* CCWGG and *B. fragilis* GGATCC. Similar to Supplementary Figure 14, but with (A) *B. fragilis* and CCWGG motif and (B) *B. fragilis* and GGATCC. All statistics are calculated as described in figure 14.

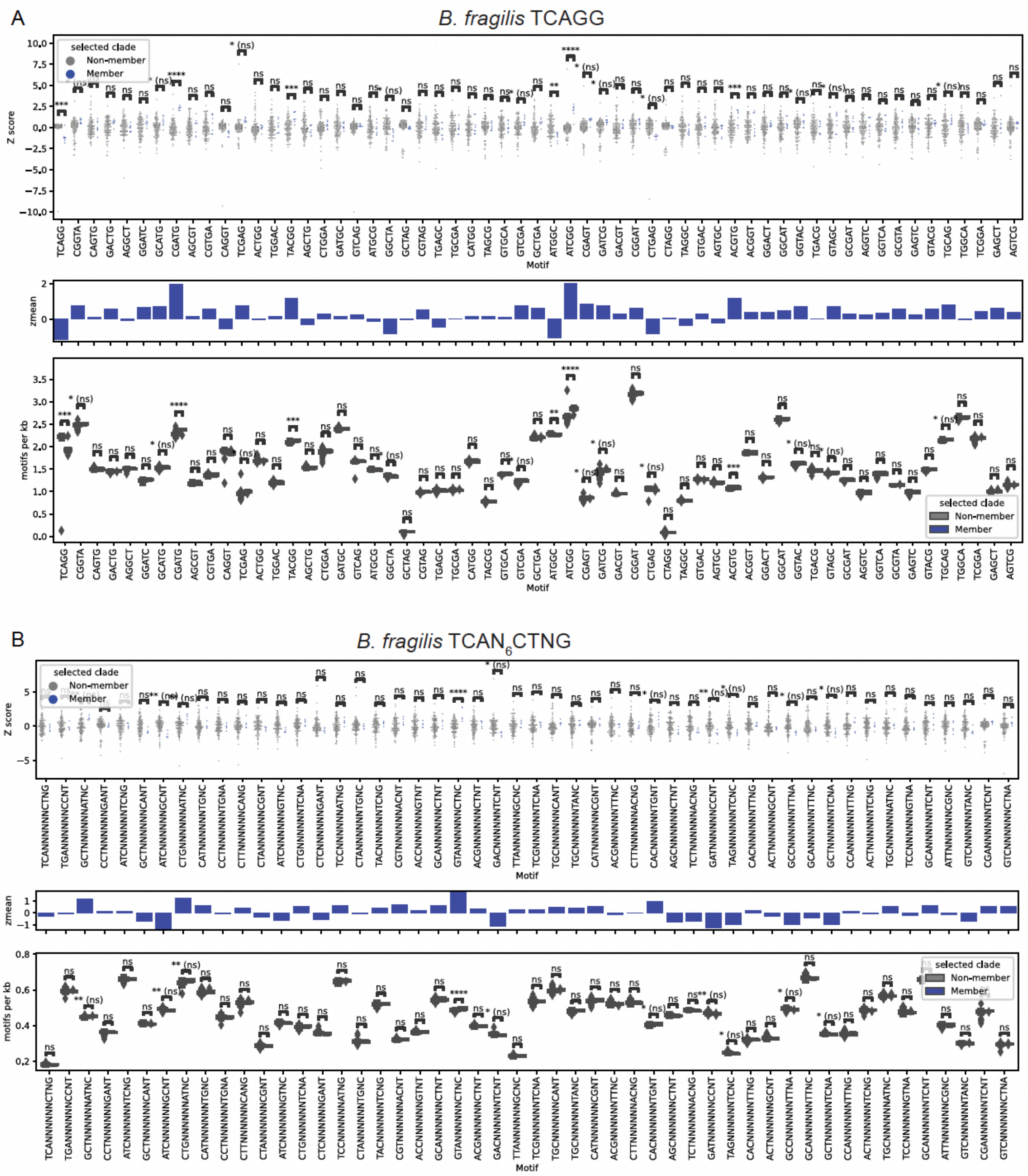

Supplementary Figure 16. Comparison of lineage-specific motif depletion to control motifs, *B. fragilis* TCAGG and *B. fragilis* TCANNNNNNCTNG. Similar to Supplementary Figure 14, but with (A) *B. fragilis* and TCAGG motif and (B) *B. fragilis* and TCANNNNNNCTNG. All statistics are calculated as described in figure 14.

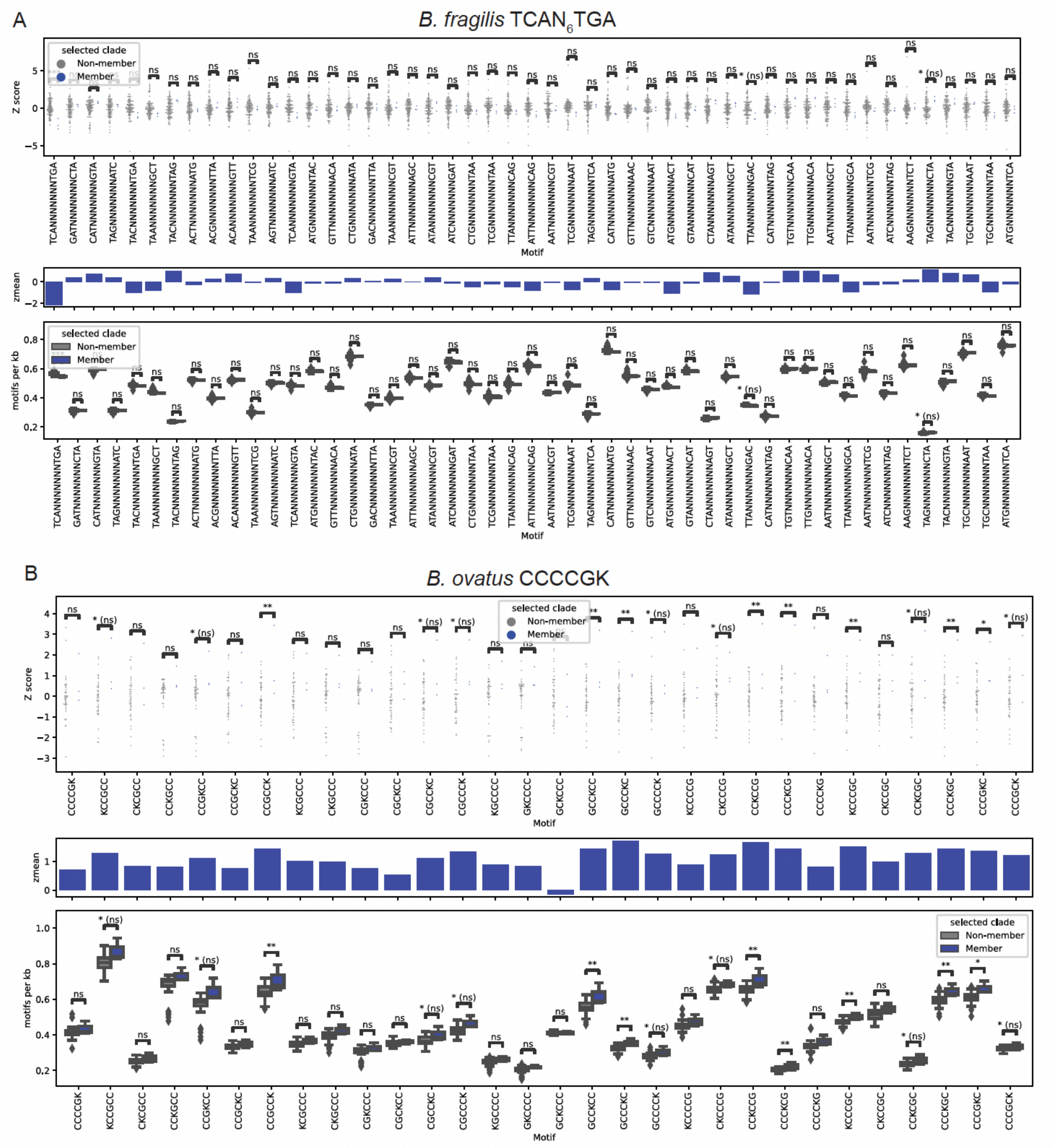

Supplementary Figure 17. Comparison of lineage-specific motif depletion to control motifs, *B. fragilis* TCANNNNNNTGA and *B. ovatus* CCCCGK. Similar to Supplementary Figure 9, but with (A) *B. fragilis* and TCANNNNNNTGA motif and (B) *B. ovatus* (n =44 genomes) and CCCCGK. All statistics are calculated as described in figure 14.

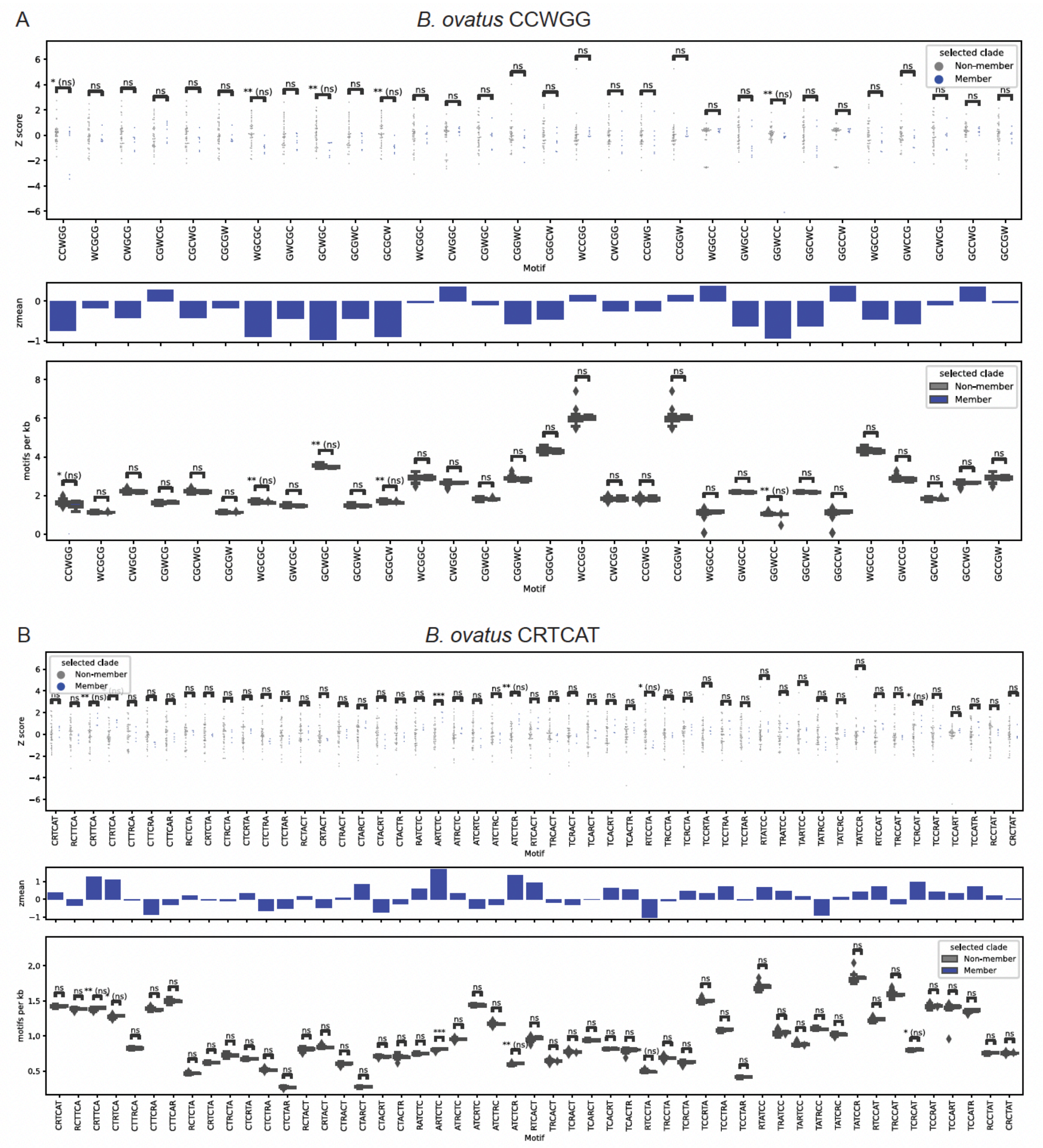

Supplementary Figure 18. Comparison of lineage-specific motif depletion to control motifs, *B. ovatus* CCWGG and *B. ovatus* CRTCAT. Similar to Supplementary Figure 14, but with (A) *B. ovatus* and CCWGG motif and (B) *B. ovatus* and CRTCAT. All statistics are calculated as described in figure 14.

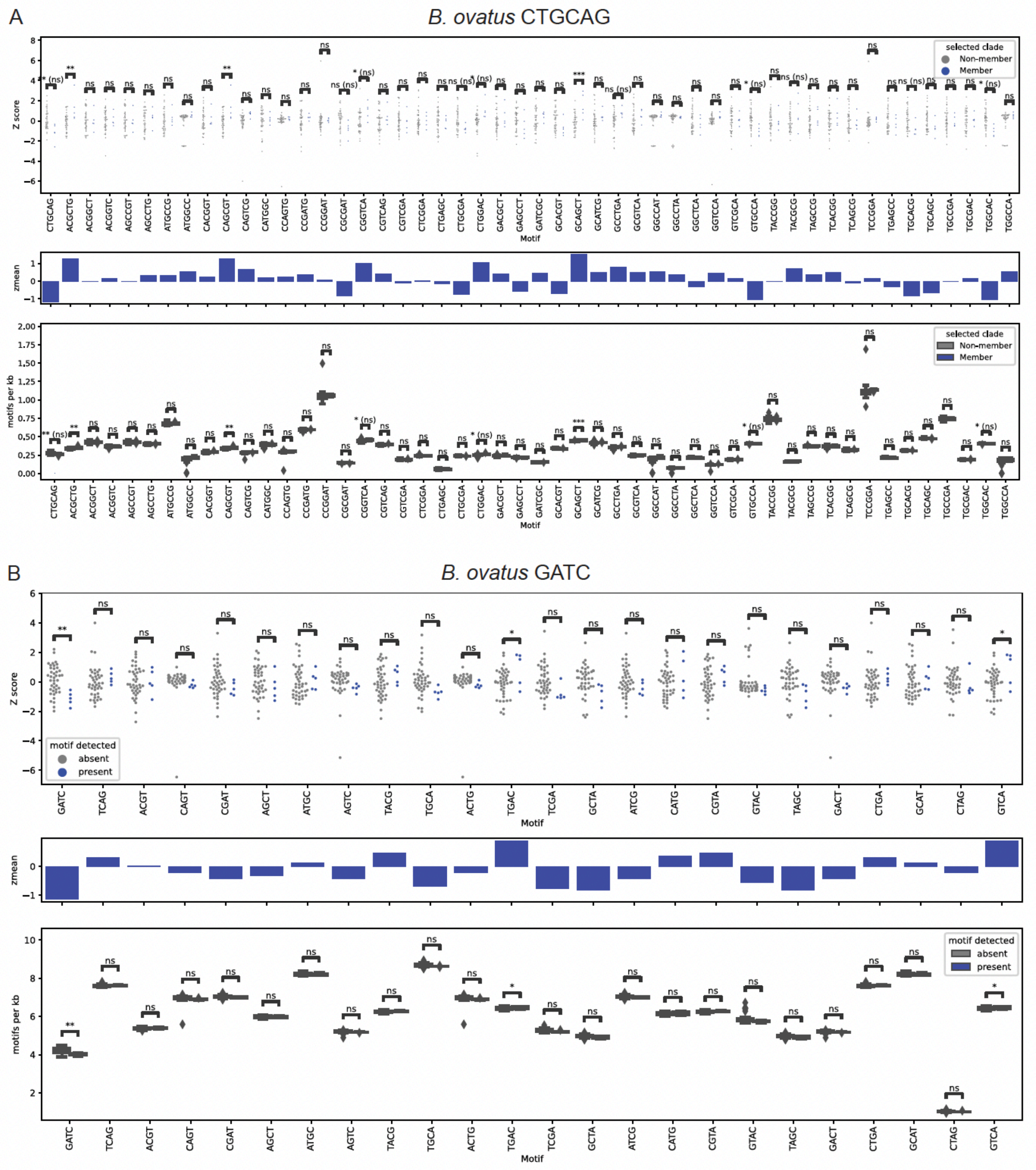

Supplementary Figure 19. Comparison of lineage-specific motif depletion to control motifs, *B. ovatus* CTGCAG and *B. ovatus* GATC. Similar to Supplementary Figure 14, but with (A) *B. ovatus* and CTGCAG motif and (B) *B. ovatus* and GATC. All statistics are calculated as described in figure 14.

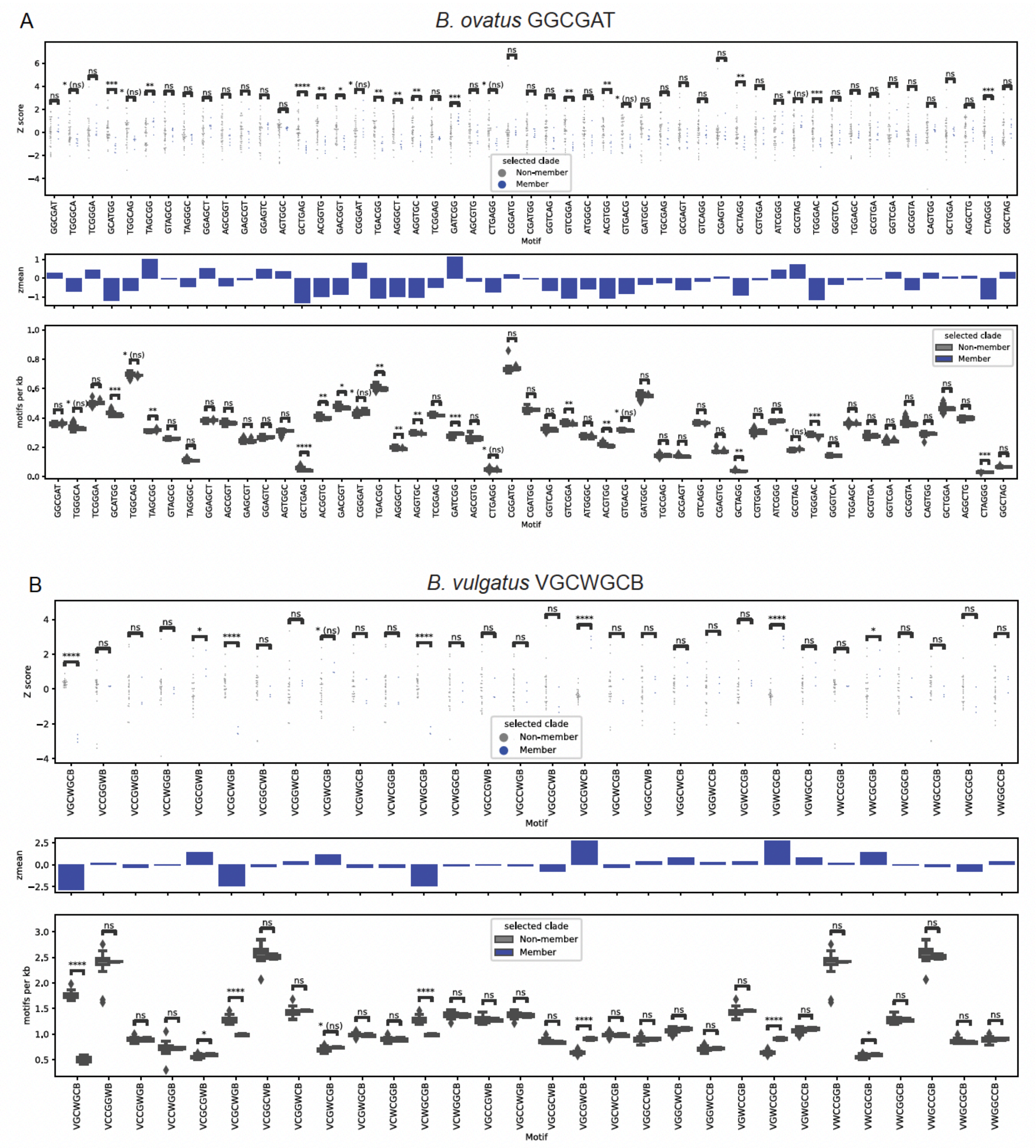

Supplementary Figure 20. Comparison of lineage-specific motif depletion to control motifs, *B. ovatus* GGCGAT and *B. vulgatus* VGCWGCB. Similar to Supplementary Figure 9, but with (A) *B. ovatus* and GGCGAT motif and (B) *B. vulgatus* (n = 28 genomes) and VGCWGCB. All statistics are calculated as described in figure 14.

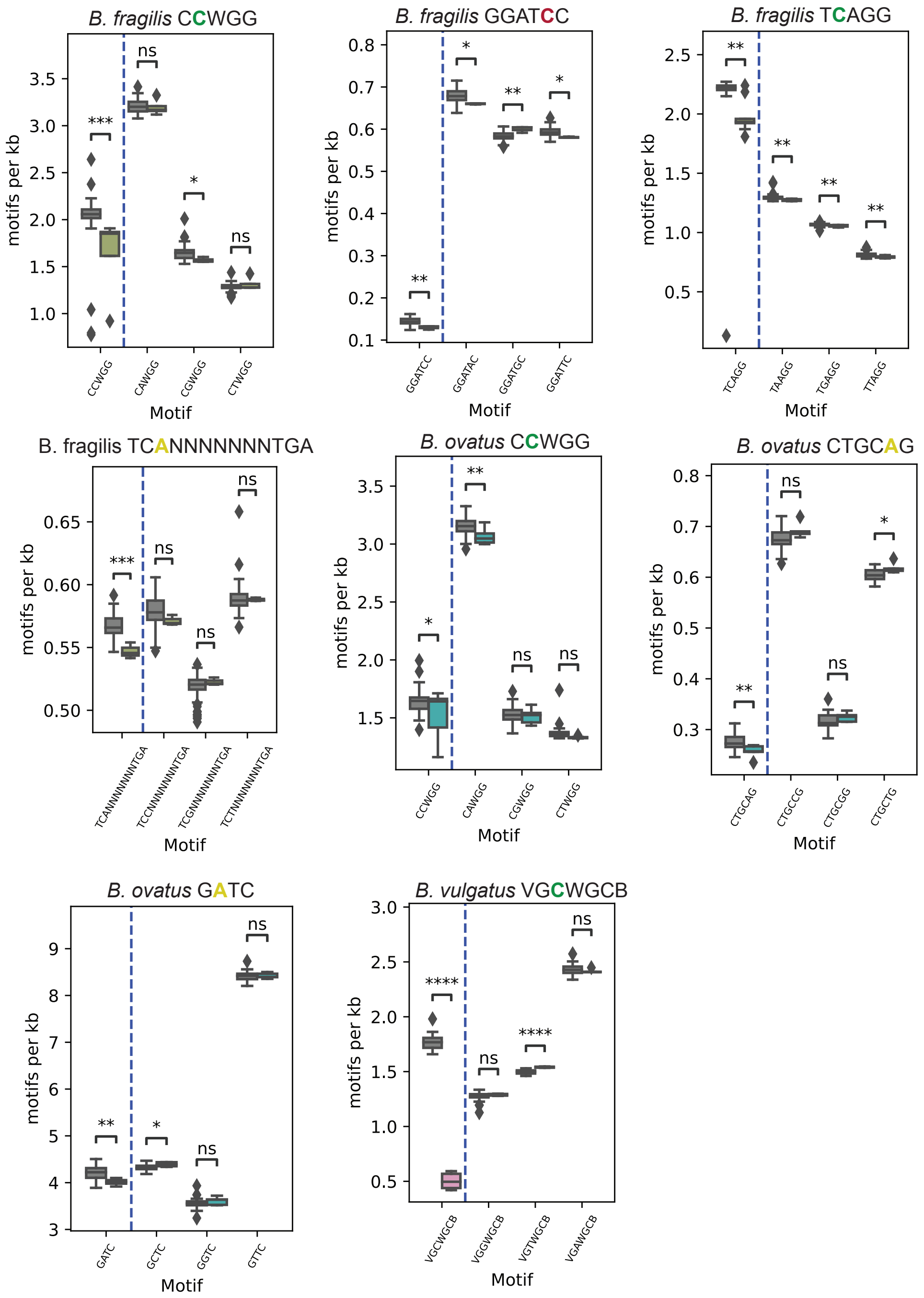

Supplementary Figure 21. Methylation Motif Target Base Mutational Analysis.

Each panel represents an analysis of DNA methylation motif abundance in a BFG sub-species lineage. Calculations for isolates in which the motif is not methylated are represented on the left in each comparison pair, and isolates in which the motif is methylated are represented on the right in each pair. The comparison left of the dotted lines displays the difference in densities of each target motif. Right of the dotted lines shows differences in motif density that would be the result of a point mutation at the methylated base of the targeted motif. (All) Boxplot boxes

show data quartiles and whiskers show 1.5X IQR with outliers drawn as diamonds. Unadjusted p values are reported as follows: *: p <= 0.05, **: p <= 0.01, ***: p <= 1e-3, ****: p <= 1e-4; “ns” indicates that the reported p value was determined to be non-significant after testing with Benjamini-Hochberg at FDR = 1%.

Supplementary Table 1. Heap's law estimates, BFG species gene families.

Species	Genomes	Heap's law: alpha
P. distasonis	29	0.7024907
B. vulgatus	34	0.718835
B. uniformis	11	0.565328
B. thetaiotaomicron	82	0.6944207
B. ovatus	55	0.7566114
B. fragilis	150	0.709654
B. faecis	23	0.8676004
B. caccae	8	1.014432

Supplementary Table 2. Comparison of PacBio and Nanodisco methylation motif calls for five isolates representing five species.

BFG-100 (*B. caccae*)

**Motif**	**Modifcation**	**Nanodisco**	**PacBio**
CC**A**TC	6mA	Y	Y
G**A**TGG	6mA	Y	Y
A**A**GNNNNNNTCC	6mA	Y	Y
GG**A**NNNNNNCTT	6mA	Y	Y
G**A**AGNNNNNNNGTT	6mA	Y	Y
A**A**CNNNNNNNCTTC	6mA	Y	Y

BFG-121 (*B. stercoris*)

**Motif**	**Modifcation**	**Nanodisco**	**PacBio**
CCN**A**G	6mA	Y	Y
G**A**TC	6mA	Y	Y
CTKM**A**G	6mA	Y	Y
TAAR**A**YC	6mA	Y	Y
CN**A**CNNNNNGGC	6mA	Y	Y
G**C**CNNNNNGTNG	6mA	Y	Y

BFG-250 (*B. cellulosilyticus*)

**Motif**	**Modifcation**	**Nanodisco**	**PacBio**
RG**A**TCY	6mA	Y	Y
AGC**A**G	6mA	Y	Y
GGTN**A**CC	6mA	Y	N
C**A**GNNNNNNTGG	6mA	Y	N
CC**A**NNNNNNTCTG	6mA	Y	N

BFG-238 (*P. distasonis*)

**Motif**	**Modifcation**	**Nanodisco**	**PacBio**
GT**A**NNNNNNGTC	6mA	Y	Y
G**A**CNNNNNNTAC	6mA	Y	Y
CC**A**GG	6mA	Y	Y
C**C**TGG	4mC	Y	Y
CTCG**A**G	6mA	Y	Y
RGAT**C**Y	6mA	Y	Y
CGTCG**A**G	6mA	N	Y
**C**GCG	5mC	Y	N

BFG-256 (*B. salyersiae*)

**Motif**	**Modifcation**	**Nanodisco**	**PacBio**
G**A**TC	6mA	Y	Y
AAG**A**CC	6mA	Y	Y
TC**A**NNNNNGTTY	6mA	Y	Y
RA**A**CNNNNNTGA	6mA	Y	Y
CC**A**NNNNNNNTGG	6mA	Y	Y
GGANG**A**C	6mA	Y	Y
**C**TAG	4mC	Y	Y
GGN**C**C	5mC	Y	N

BFG-1 (*B. fragilis*)

**Motif**	**Modifcation**	**Nanodisco**	**PacBio**
CTC**A**T	6mA	Y	Y
CCA**A**G	6mA	Y	Y
**A**GCNNNNRTTG	6mA	Y	Y
CA**A**YNNNNGCT	6mA	Y	Y

Supplementary Table 3. Heap's law estimates, DNA methylation motifs in BFG and other species.

Species	Genomes	Heap's law: alpha
P. distasonis	23	0.1927405
B. vulgatus	27	0.2161848
B. thetaiotaomicron	53	0.2115285
B. ovatus	43	0.2059093
B. fragilis	107	0.2320848
C. difficile	36	0.70377
Bifi. Breve	31	0.5158546

Supplementary Table 4. DNA methylation control motif enrichment/depletion summary

Target Motif	Total control motifs	Controls significantly enriched	Controls significantly depleted	Controls significantly enriched %	Controls significantly depleted %
CCANNNNNNTCG	48	1	0	2.1	0
CCCCGK	29	8	0	27.6	0
CCNGG	16	6	0	37.5	0
CCWGG (B. fragilis)	29	0	4	0	13.8
CCWGG (B. ovatus)	29	0	0	0	0
CRTCAT	50	1	0	2	0
CTGCAG	49	0	0	0	0
GGATCC	48	0	1	0	2.1
GGCGAT	50	2	12	4	24
TCAGG	58	4	1	6.9	1.7
TCANNNNNNCTNG	49	1	0	2.0	0
TCANNNNNNNTGA	48	0	0	0	0
VGCWGCB	29	4	2	13.8	6.9
TOTAL	532	27	20	5.08	3.8
