# Supplementary material for: Roving methyltransferases generate a mosaic epigenetic landscape and influence evolution in Bacteroides fragilis group
Source: Nat Commun. 2023 Jul 10;14:4082. doi: 10.1038/s41467-023-39892-6 (PMC10333322; doi:10.1038/s41467-023-39892-6)
Supplement: Supplementary file 4 — Description of Additional Supplementary Files [file 41467_2023_39892_MOESM4_ESM.docx]

**Description of Additional Supplementary Files**

**File Name: Supplementary Data 1
Description:** Table with assembly statistics of each BFG isolate genome.

**File Name: Supplementary Data 2
Description:** BFG isolate and GenBank reference metadata/taxonomy used to decorate Figure 1.

**File Name: Supplementary Data 3
Description:** All accessory region sequences over 3 kb from BFG isolate genomes and GenBank reference genomes in fasta format.

**File Name: Supplementary Data 4
Description:** All circular episomes/plasmids from BFG isolate genomes in fasta format.

**File Name: Supplementary Data 5
Description:** All predicted DNA methylase gene protein sequences, fasta format.

**File Name: Supplementary Data 6
Description:** All predicted DNA methylase gene family clusters at 80% AAI and 80% AF.

**File Name: Supplementary Data 7
Description:** Identified putative methyltransferases in isolate BFG-632 in fasta format.

**File Name: Supplementary Data 8
Description:** All predicted prophage region sequences BFG isolate genomes and GenBank reference genomes in fasta format.

**File Name: Supplementary Data 9
Description:** All predicted prophage virus OTU clusters at 95% ANI and 85% AF.

**File Name: Supplementary Data 10
Description:** All predicted prophage sequence quality summary from CheckV.

**File Name: Supplementary Data 11
Description:** Complete data for Figure 5. All predicted methylation motifs in all studied species.

**File Name: Supplementary Data 12
Description:** Abricate (AMR gene) detection results from BFG isolate genomes (proovframe correction) (80% minid, 80% mincov).

**File Name: Supplementary Data 13
Description:** HMMs of conjugative machinery protein domains, manually selected from PFAM, formatted for hmmer3.

**File Name: Supplementary Data 14
Description:** Text files with names of CDD models for detecting conjugative machinery, relaxases, replicases, integrases, and transposases from a RPS-BLAST search.
